# Supplementary material for: Cell-type deconvolution of bulk-blood RNA-seq reveals biological insights into neuropsychiatric disorders
Source: Am J Hum Genet. 2024 Feb 1;111(2):323–37. doi: 10.1016/j.ajhg.2023.12.018 (PMC10870131; doi:10.1016/j.ajhg.2023.12.018)
Supplement: Document S1. Figures S1–S8 [file mmc1.pdf]

The American Journal of Human Genetics, Volume *111*

Supplemental information

Cell-type deconvolution of bulk-blood RNA-seq reveals biological insights into neuropsychiatric disorders

Toni Boltz, Tommer Schwarz, Merel Bot, Kangcheng Hou, Christa Caggiano, Sandra Lapinska, Chenda Duan, Marco P. Boks, Rene S. Kahn, Noah Zaitlen, Bogdan Pasaniuc, and Roel Ophoff

Supplemental Figures

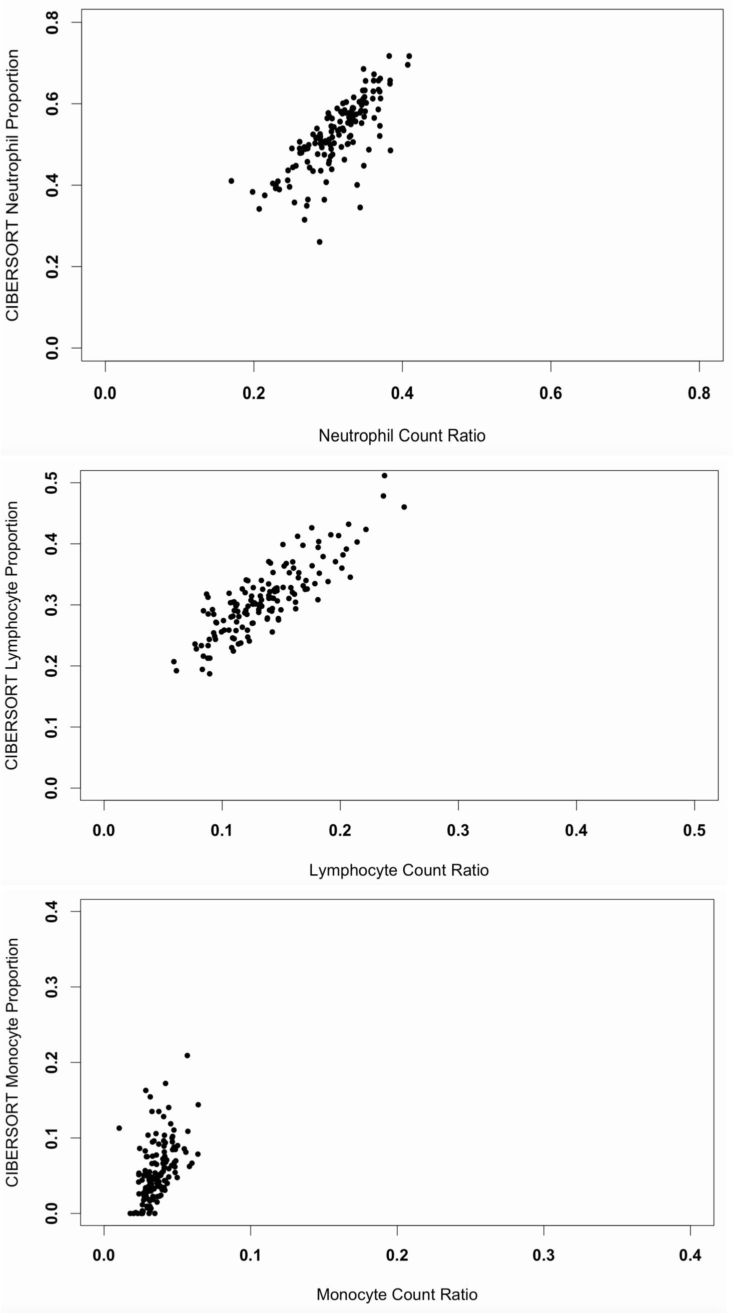

Figure S1: Scatterplots of CIBERSORTx-estimated cell type proportions vs complete blood count proportions. We find generally high concordance between computationally estimated and measured ground truth cell type proportions using a subset of our cohort. Pearson's correlation R2 for neutrophils = 0.76, for lymphocytes = 0.85, for monocytes = 0.48.

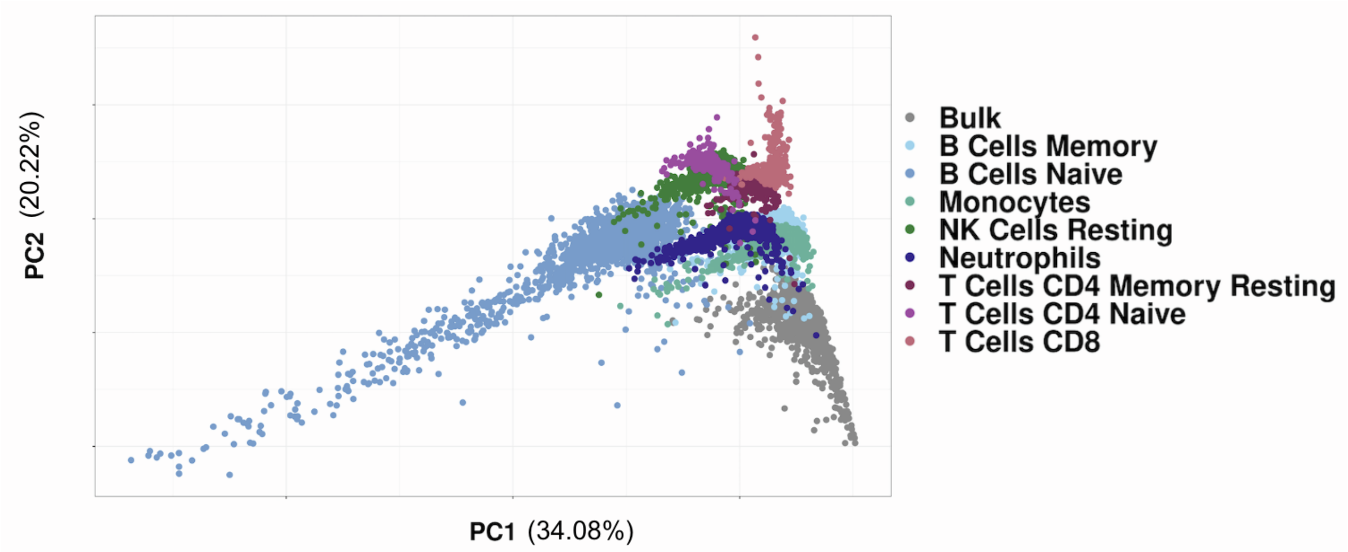

Figure S2: Principal component analysis of cell type expression. Each point represents an individual's gene expression profile, colored by the specific context, thus each individual is represented nine times on this plot.

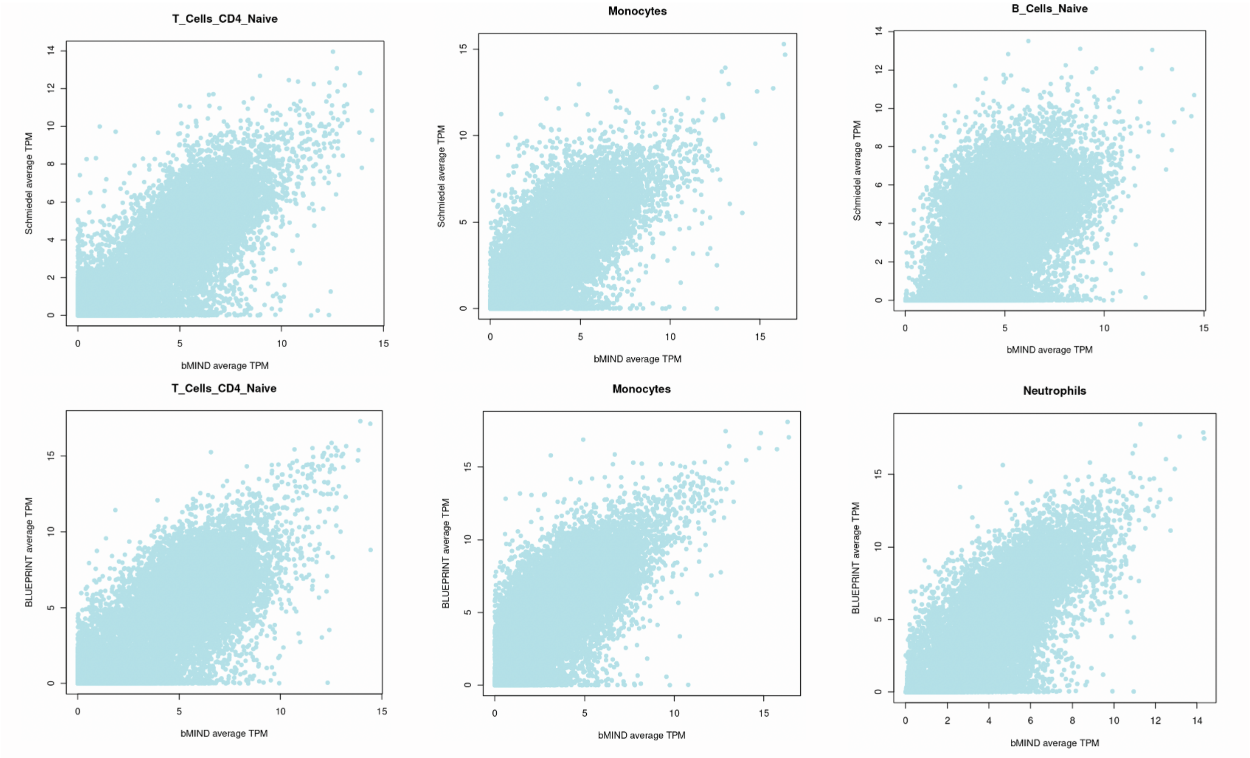

Figure S3: Scatterplots of expression estimated from bulk vs single-cell reference datasets.

Using two scRNA-Seq datasets as references (from the eQTLGen[1] study using BLUEPRINT[2] Consortium and Schmiedel et. al. (2018)[3] datasets, available online at: https://github.com/eQTL-Catalogue/eQTL-Catalogue-resources/blob/master/tabix/tabix_ftp_paths.tsv) we compare the median TPM values for protein coding genes using both scRNA-Seq and computationally deconvoluted bulk RNA-Seq.

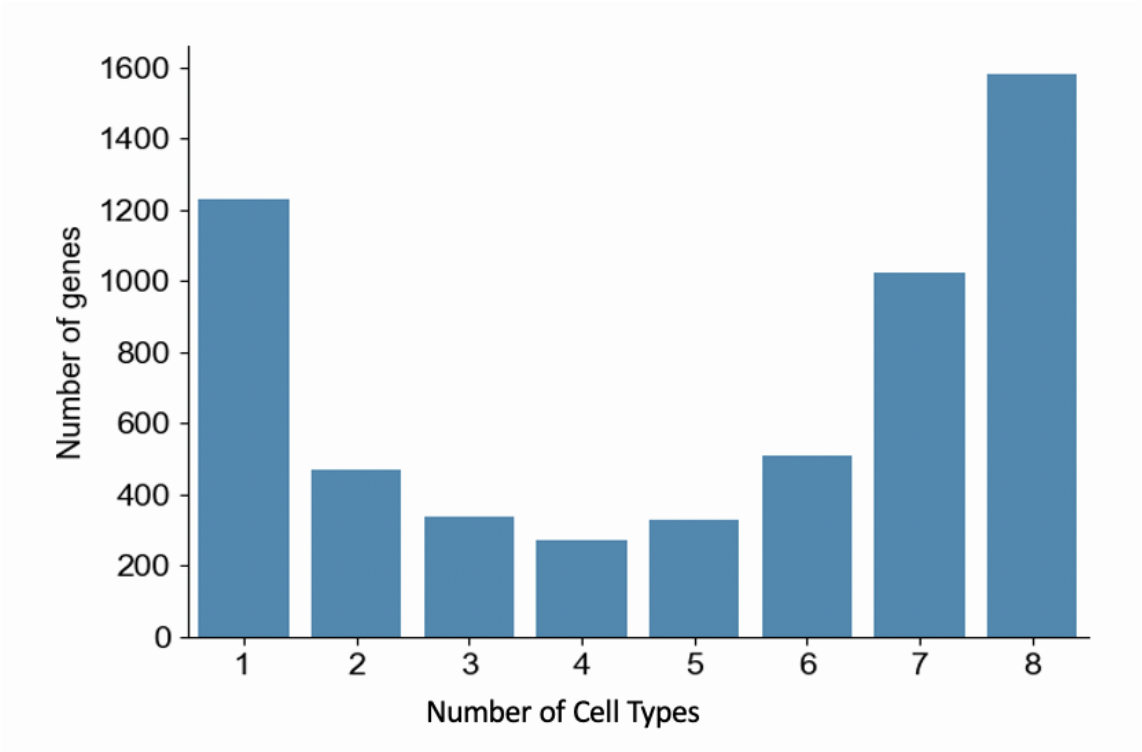

Figure S4: Distribution of shared eGenes across cell type contexts.

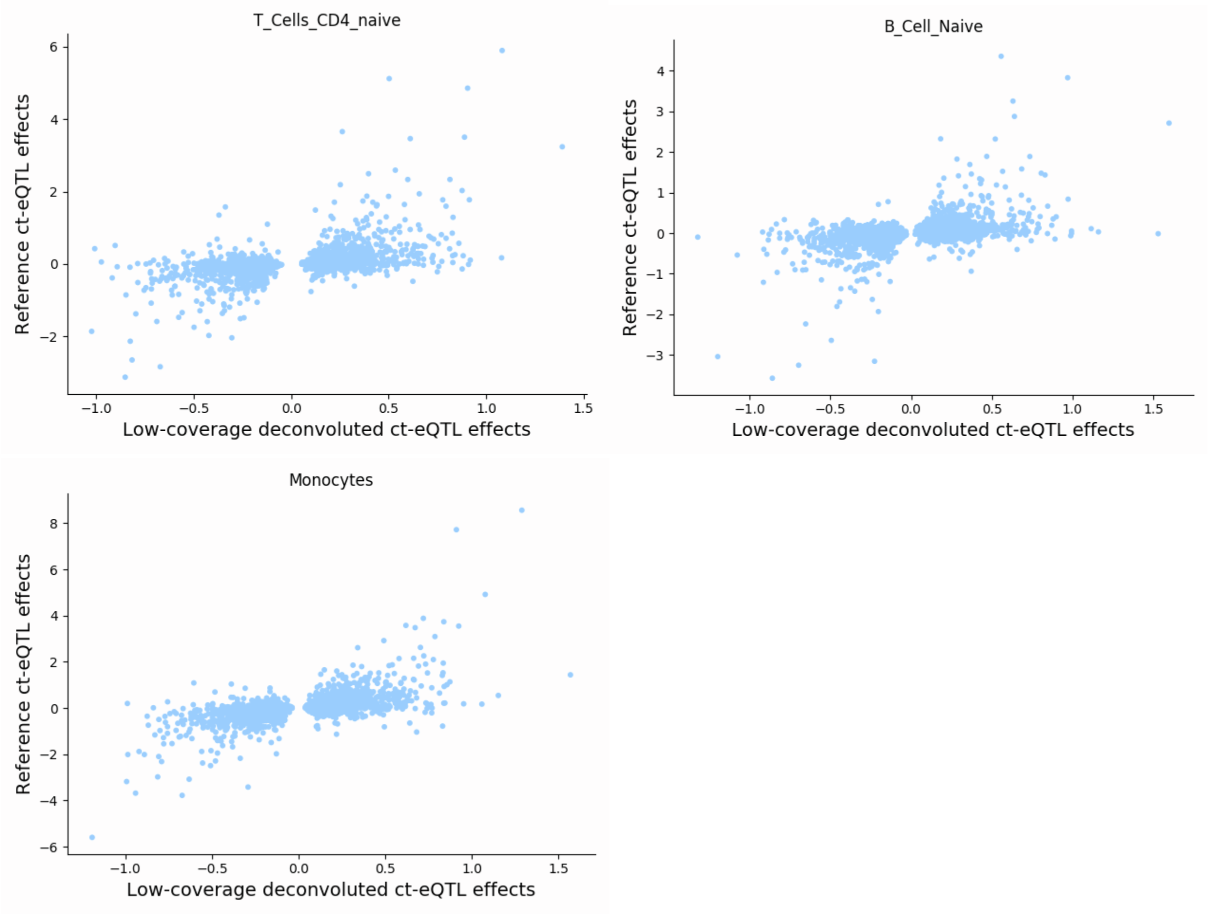

Figure S5: Effect size correlations between reference single cell eQTL and the deconvoluted eQTL. T cells CD4 naive R2 = 0.27; B cells naive R2 = 0.22; Monocytes R2 = 0.36.

A.

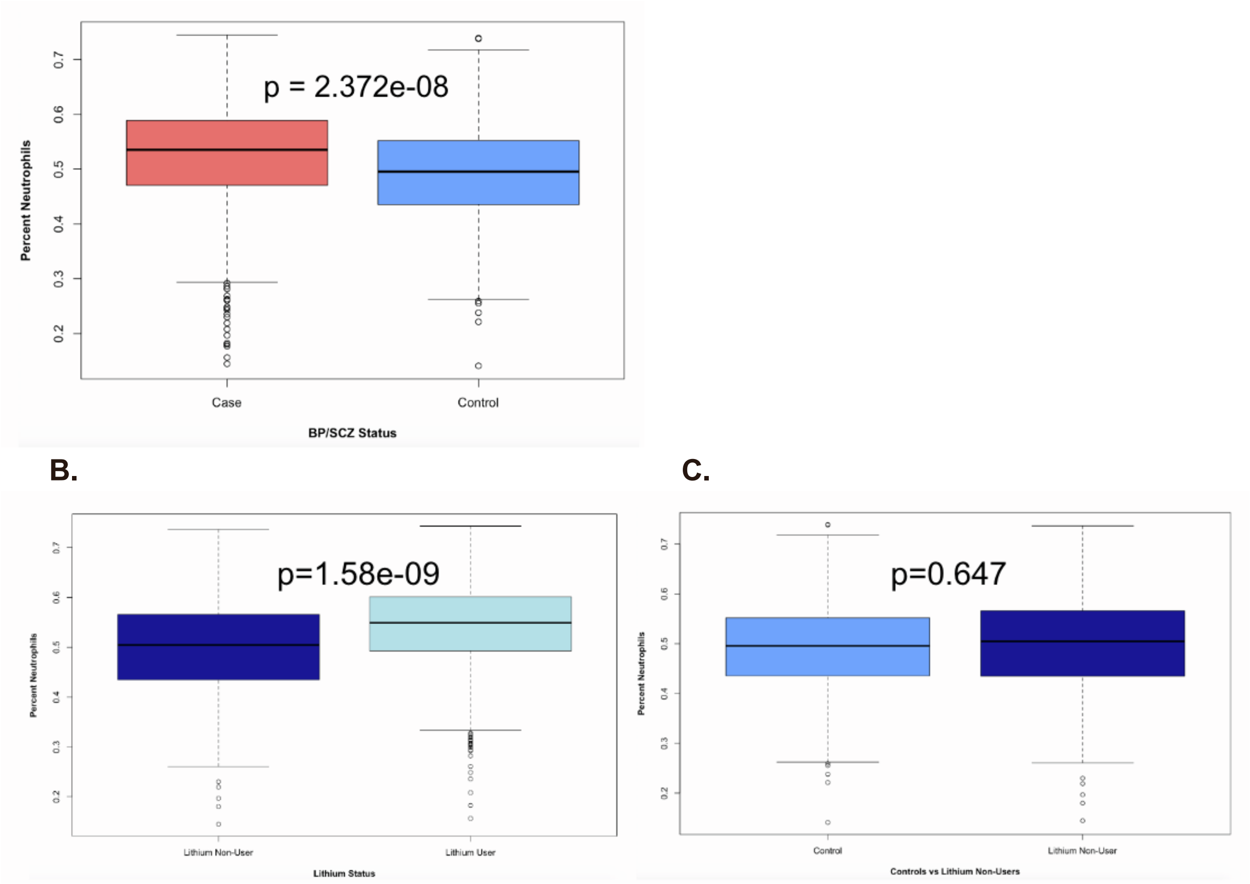

Figure S6: Neutrophil count elevated for lithium users.

A. Difference in neutrophil proportion (after accounting for covariates including age, sex, RIN, and RNA concentration) between BP/SCZ cases and controls.
B. Difference in neutrophil proportion between lithium users and non-users (after accounting for covariates), only within BP cases.
C. Difference in neutrophil proportion between lithium non-users and controls (after accounting for covariates).

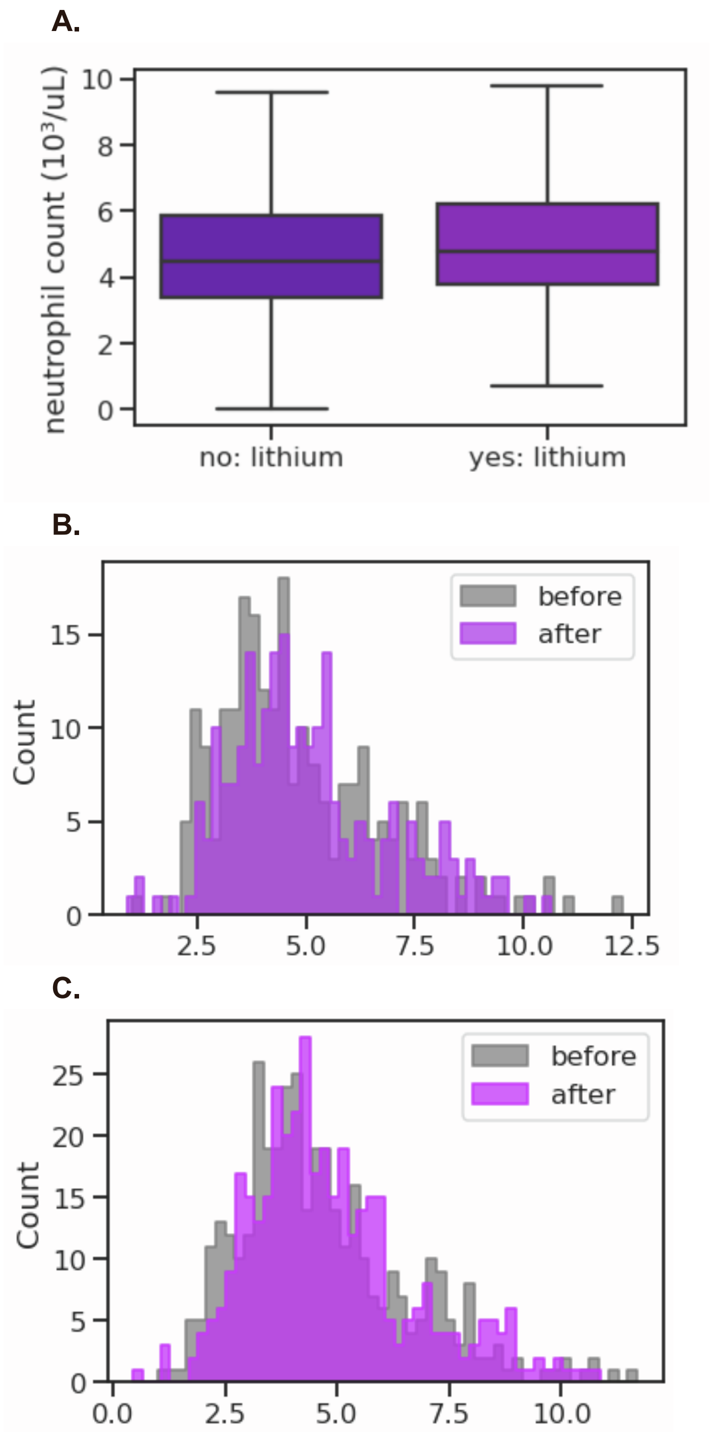

Figure S7: Neutrophil count elevated for lithium users in UCLA ATLAS.

A. Median neutrophil count across self-reported European individuals (N=7,350), with covariate correction for age and sex (p=2.09e-7).
B. Neutrophil count distribution across self-reported European individuals (n=229) before and after lithium prescription (Wilcoxon p=0.2).
C. Neutrophil count distribution across individuals (n=376, all ancestries) before and after lithium prescription (Wilcoxon p=0.0228).

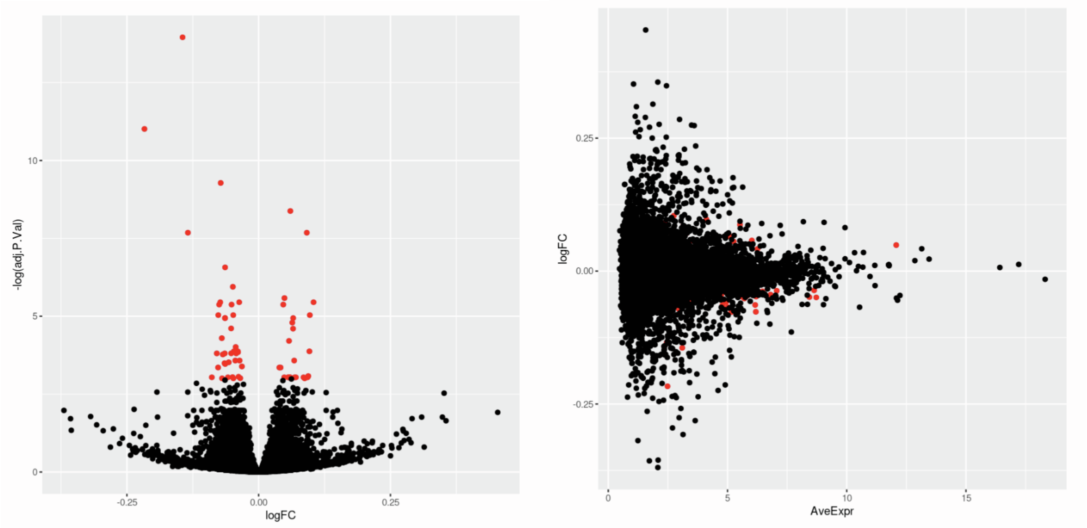

Figure S8: **Differential gene expression results for BP or SCZ cases vs controls:** (left) Volcano plot which highlights differentially expressed genes (FDR < 0.05) in red (N=64 total differentially expressed genes). (right) Average expression of each gene vs the log fold change (logFC) of each gene, with differentially expressed genes highlighted in red.

Supplemental References

1. Kerimov, N., Hayhurst, J.D., Peikova, K., Manning, J.R., Walter, P., Kolberg, L., Samoviča, M., Sakthivel, M.P., Kuzmin, I., Trevanion, S.J., et al. (2021). A compendium of uniformly processed human gene expression and splicing quantitative trait loci. Nat. Genet. *53*, 1290–1299.

2. Chen, L., Ge, B., Casale, F.P., Vasquez, L., Kwan, T., Garrido-Martín, D., Watt, S., Yan, Y., Kundu, K., Ecker, S., et al. (2016). Genetic Drivers of Epigenetic and Transcriptional Variation in Human Immune Cells. Cell *167*, 1398–1414.e24.

3. Schmiedel, B.J., Gonzalez-Colin, C., Fajardo, V., Rocha, J., Madrigal, A., Ramírez-Suástegui, C., Bhattacharyya, S., Simon, H., Greenbaum, J.A., Peters, B., et al. (2022). Single-cell eQTL analysis of activated T cell subsets reveals activation and cell type-dependent effects of disease-risk variants. Science Immunology 7(68).
