# Supplementary material for: Cell-type deconvolution of bulk-blood RNA-seq reveals biological insights into neuropsychiatric disorders
Source: Am J Hum Genet. 2024 Feb 1;111(2):323–37. doi: 10.1016/j.ajhg.2023.12.018 (PMC10870131; doi:10.1016/j.ajhg.2023.12.018)
Supplement: Document S2. Article plus supplemental information [file mmc17.pdf]

# Cell-type deconvolution of bulk-blood RNA-seq reveals biological insights into neuropsychiatric disorders

## Graphical abstract

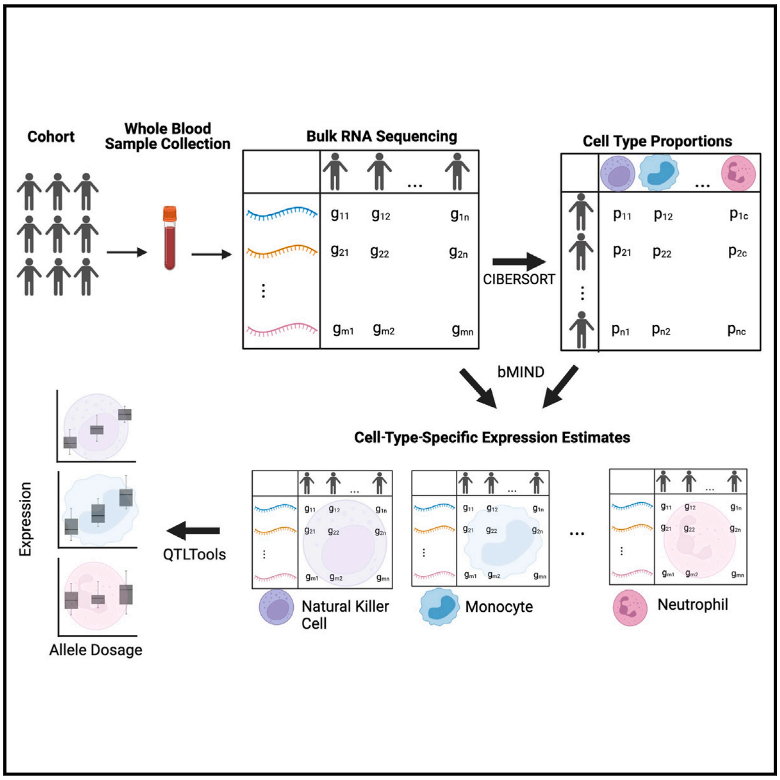



## Authors

Toni Boltz, Tommer Schwarz, Merel Bot, ..., Noah Zaitlen, Bogdan Pasaniuc, Roel Ophoff

## Correspondence

tboltz@g.ucla.edu (T.B.), ophoff@ucla.edu (R.O.)



**Methods aimed at deconvolving cell-type-specific estimates of bulk gene expression offer a cost-effective way to investigate cell-type-specific eQTLs. Our study suggests that such computational approaches to a large sample with blood-based bulk RNA-seq significantly increase the opportunity to interpret the underlying biology of brain-specific disease susceptibility.**


Boltz et al., 2024, The American Journal of Human Genetics *111*, 323–337
February 1, 2024
https://doi.org/10.1016/j.ajhg.2023.12.018

# Cell-type deconvolution of bulk-blood RNA-seq reveals biological insights into neuropsychiatric disorders

Toni Boltz,[1,11,*] Tommer Schwarz,[2,11] Merel Bot,[3] Kangcheng Hou,[2] Christa Caggiano,[2] Sandra Lapinska,[2] Chenda Duan,[4] Marco P. Boks,[5] Rene S. Kahn,[5,6] Noah Zaitlen,[2,7] Bogdan Pasaniuc,[1,2,8,9] and Roel Ophoff[1,2,3,10,*]

## Summary

Genome-wide association studies (GWASs) have uncovered susceptibility loci associated with psychiatric disorders such as bipolar disorder (BP) and schizophrenia (SCZ). However, most of these loci are in non-coding regions of the genome, and the causal mechanisms of the link between genetic variation and disease risk is unknown. Expression quantitative trait locus (eQTL) analysis of bulk tissue is a common approach used for deciphering underlying mechanisms, although this can obscure cell-type-specific signals and thus mask trait-relevant mechanisms. Although single-cell sequencing can be prohibitively expensive in large cohorts, computationally inferred cell-type proportions and cell-type gene expression estimates have the potential to overcome these problems and advance mechanistic studies. Using bulk RNA-seq from 1,730 samples derived from whole blood in a cohort ascertained from individuals with BP and SCZ, this study estimated cell-type proportions and their relation with disease status and medication. For each cell type, we found between 2,875 and 4,629 eGenes (genes with an associated eQTL), including 1,211 that are not found on the basis of bulk expression alone. We performed a colocalization test between cell-type eQTLs and various traits and identified hundreds of associations that occur between cell-type eQTLs and GWASs but that are not detected in bulk eQTLs. Finally, we investigated the effects of lithium use on the regulation of cell-type expression loci and found examples of genes that are differentially regulated according to lithium use. Our study suggests that applying computational methods to large bulk RNA-seq datasets of non-brain tissue can identify disease-relevant, cell-type-specific biology of psychiatric disorders and psychiatric medication.

## Introduction

One limitation of standard eQTL studies is that they generally use expression estimates from bulk tissue.[1,2] Although this is informative, it has been shown that there are many cell-type-specific mechanisms driving biology,[3,4] and these can be missed when one looks at a collection of many cell types. In recent years, single-cell RNA-seq has allowed for the profiling of the gene expression of an individual cell, giving us a clearer picture of cell-type gene expression. However, single-cell RNA-seq experiments are considerably more expensive than bulk RNA-seq.[5] To leverage the advantages of each of these approaches, we can estimate cell-type gene expression from bulk RNA-seq expression.

Computational methods for analyzing bulk gene-expression data have the potential for being advantageous in some applications because it is possible to obtain much larger sample sizes by using bulk RNA-seq instead of single-cell RNA-seq. While most single-cell RNA-seq studies have sample sizes in the range of several hundreds of cells from a small number of individuals, leveraging low-coverage bulk RNA-seq allows us to obtain samples from hundreds to thousands of subjects.[6] We used the low-coverage whole-blood RNA-seq dataset (dbGAP: phs002856.v1) with approximately 5.9 million reads on average per sample, as described in Schwarz et al.,[6] as the primary dataset for analysis of cell-type deconvolution in this study.

There exist many methods[7–9] of estimating cell-type expression from bulk RNA-seq, including methods employing gene-by-environment interaction models[2] such as Decon-eQTL[10] and imputation-based methods, such as CIBERSORTx[11] and bMIND.[12] In this study, we elected to use CIBERSORTx[11] and bMIND[12] to estimate cell-type proportions and cell-type expression, respectively. CIBERSORTx has been previously shown[6] to perform similarly in computing cell-type proportions at both lower and moderate RNA-seq coverage levels. Although CIBERSORTx and other methods[13–15] are also able to impute cell-type gene-expression data, they require a single-cell RNA-seq

[1]Department of Human Genetics, David Geffen School of Medicine, University of California Los Angeles, Los Angeles, CA, USA; [2]Bioinformatics Interdepartmental Program, University of California Los Angeles, Los Angeles, CA, USA; [3]Center for Neurobehavioral Genetics, Semel Institute for Neuroscience and Human Behavior, University of California, Los Angeles, Los Angeles, CA, USA; [4]Department of Computer Science, University of California, Los Angeles, Los Angeles, CA, USA; [5]Department of Psychiatry, Brain Center, University Medical Center Utrecht, University Utrecht, Utrecht, the Netherlands; [6]Department of Psychiatry, Icahn School of Medicine, Mount Sinai, NY, USA; [7]Department of Neurology, University of California Los Angeles, Los Angeles, Los Angeles, CA, USA; [8]Department of Computational Medicine, David Geffen School of Medicine, University of California Los Angeles, Los Angeles, CA, USA; [9]Department of Pathology and Laboratory Medicine, David Geffen School of Medicine, University of California Los Angeles, Los Angeles, CA, USA; [10]Department of Psychiatry, Erasmus University Medical Center, Rotterdam, the Netherlands
[11]These authors contributed equally
*Correspondence: tboltz@g.ucla.edu (T.B.), ophoff@ucla.edu (R.O.)
https://doi.org/10.1016/j.ajhg.2023.12.018.
This is an open access article under the CC BY license (http://creativecommons.org/licenses/by/4.0/).

reference dataset, ideally in a matched subset of individuals, for all the cell types of interest across all genes of interest. The motivation of this study is to evaluate the use of bulk blood gene expression for cell-type-specific analysis, without the input of matched single-cell sequencing data.

Associations between immune-related traits and neuropsychiatric disorders have been previously reported,[16] and we hypothesized that using blood-based expression can provide relevant information regarding the biology of such disorders.[17–19] Although brain tissue would be the most relevant for studying mechanisms of neuropsychiatric disorders, recent research has shown that blood-derived eQTL analysis replicates more than half of the eQTL found in brain tissue.[20] Similarly, previous work[17] has found a high correlation ($R^2 = 0.7$) between blood- and brain-derived eQTL effect sizes, suggesting that whole blood can be a useful proxy when brain tissue is not available, although with the caveat that some brain-specific biology will not be detected in blood.

In this work we used cell-type deconvolution methods to derive cell-type-specific estimates for gene expression from bulk-blood RNA-seq, specifically within a cohort including individuals diagnosed with psychiatric disorders and controls of European ancestry. We used these results to conduct cell-type *cis*-eQTL analyses and compared the shared and unique cell-type associations. We show that these cell-type eQTL results derived from deconvoluted bulk RNA-seq are consistent with eQTLs from scRNA-seq. We performed colocalization analysis to find loci driving GWAS associations in either neuropsychiatric or blood-based traits and cell-type gene expression. We went on to identify several examples of "opposite-effect" eQTLs, where a cell-type eQTL signal demonstrates gene expression regulation in the opposite direction from that observed in a bulk eQTL study. Finally, we explored the effects of lithium use[21] on cell-type expression and identified several cases of lithium-SNP interaction dictating the presence of an eQTL.

## Subjects and methods

### Cohort description

The samples included are from a study with individuals ascertained for bipolar disorder (BP) or schizophrenia (SCZ). The cohort consists of 1,045 individuals with BP, 84 individuals with SCZ, and 601 controls with whole-blood RNA-seq and corresponding genotypes (n = 1,730 after exclusion of first-degree relatives) included for all individuals. Data were generated according to protocols approved by the respective local ethics committees: the Medical Ethical Review Board at University Medical Center Utrecht and the Institutional Review Board (IRB) at University of California Los Angeles. Informed consent was obtained from all subjects.

### Bulk RNA sequencing

Bulk RNA sequencing was performed at the UCLA Neurogenomics Core according to the TruSeq Stranded plus rRNA and GlobinZero library preparation method, as described previously.[6] We used FASTQC[22] (see web resources) to visually inspect the read quality from the lower-coverage whole-blood RNA-seq (5.9M reads/sample). We then used kallisto[23] to pseudoalign reads to the GRCh37 gencode transcriptome (v. 33) and quantify estimates for transcript expression. We aggregated transcript counts to obtain gene-level read counts by using scripts from the GTEx consortium (https://github.com/broadinstitute/gtex-pipeline).

### Genotyping pipeline

Genotypes for the individuals included in the cohort were obtained from the following platforms: OmniExpressExome (n = 816), Psych Chip (n = 522), COEX (n = 162), Illumina550 (n = 19), and Global Screening Array (n = 211). Given that the SNP-genotype data came from numerous studies, the number of overlapping SNPs across all platforms was <80k, prompting us to perform imputation separately for each genotyping platform, as previously described in Schwarz et al. 2022.[6] In brief, variants were first filtered for Hardy-Weinberg equilibrium p value $< 1.0 \times 10^{-6}$ for control individuals and p value $< 1.0 \times 10^{-10}$ for affected individuals, with minor-allele frequency (MAF) > 0.01. Then, we used the 1000 Genomes Project phase 3 reference panel[24] to impute genotypes by chromosome by using RICOPILI v.1[25] separately per genotyping platform, then subsequently merging platforms. We assessed imputation quality by filtering variants where genotype probability >0.8 and INFO score >0.1. We restricted it to only autosomal chromosomes because of the sex-chromosome dosage, as commonly done.[26] All rsIDs referenced throughout the manuscript are referring to reference genome build GRCh37.

### Cell-type proportion estimation

We estimated the proportion of cell types of the bulk whole-blood RNA-seq datasets by using CIBERSORTx and applied batch correction and used LM22 signature matrix as the reference gene expression profile. The LM22 signature matrix uses 547 genes to distinguish between 22 human hematopoietic cell phenotypes (downloaded from: https://cibersortx.stanford.edu/download.php), although here we restrict these to 8 cell types with proportions >0.02.

Complete blood count (CBC) lab tests from the clinic were provided for a subset of the cohort (n = 143), providing us ground truth measures (in units of $10^9$ cells per liter) for neutrophils, lymphocytes, monocytes, basophils, and eosinophils. To make the counts comparable to the proportions output by CIBERSORTx, we divided the counts of the cell type of interest by the sum of counts across all cell types in an individual, providing the count ratio shown in Figure S1.

### Cell-type expression estimation

We log2-transformed the matrix of bulk TPM values before inputting these values into bMIND because the largest expression value was greater than 50 TPM. Using the cell-type proportions derived from CIBERSORTx in conjunction with these log-transformed bulk expression measures, we used bMIND to derive cell-type expression estimates, with flag np = TRUE.

Imputed gene expression can vary on the basis of the method chosen, although bMIND was chosen over CIBERSORTx for gene expression imputation because of its improved computational efficiency and improved average $R^2$ for genes when correlated against snRNA-seq gene expression.[12]

## *cis*-eQTL mapping

Using output from bMIND, we transformed expression estimates from log2(TPM) to counts by using sequencing libraries, specifically restricted to sufficiently expressed genes (estimated count >1.0 in 40% of individuals). We then standardized expression estimates (mean = 0) and performed *cis*-eQTL analysis mapping with QTLTools,[27] by using a defined window of 1 Mb both up and downstream of every gene's TSS, for sufficiently expressed genes (TPM > 0.1 in 20% of individuals). Covariates for the first 50 expression PCs, age, sex, RNA concentration, and RNA integrity values were included. We ran the eQTL analysis in permutation pass mode (1,000 permutations) and performed multiple testing corrections by using the q value FDR (false discovery rate) procedure; we correct at 5% unless otherwise specified. We then restricted associations to the top (or leading) SNP per eGene.

We also ran Decon-eQTL[10] to compare the results of using a cell-type proportion interaction model versus gene expression imputation followed by QTL analysis. Fisher's exact test showed a significant overlap in the eGenes identified by either method when we considered nominally significant ($p < 0.05$) results, suggesting that these methods each detect similar cell-type-specific signals from bulk expression data. Correcting for the multiple testing, however, resulted in many fewer eGenes' reaching the threshold for significance with Decon-eQTL (Table S1); thus, we proceeded with the imputation-based method.

## Replication rates with reference eQTL datasets

Following the methodology used previously in cell-type eQTL studies,[28,29] We used the qvalue()[30] function in R to estimate Storey's $\pi_1$. Namely, we took the eQTL called as FDR-significant in each reference dataset and pulled the nominal p values of the corresponding eQTL in each computationally derived cell type. We set lambda to the maximum p value within our eQTL results subset and estimated the $\pi_0$, then computed the replication rate as $\pi_1 = 1 - \pi_0$.

Table S2 provides the replication rates of p values between the bulk-detectable eQTL and the bMIND+fastQTL-detected eQTL. Note that for neutrophils, the R function for the estimation of $\pi 1$ is unstable because the solution lies near the $\pi 1 = 1$ boundary. To verify that this was the case, we added null p values to the data by randomly sampling the uniform (0,1) distribution (at a 10% fraction of the number of gene-SNP pairs). $\pi 1$ was estimated to be approximately 1 − (fraction of null p values), here $(1 - 0.10) = 0.9$.

## TWAS and colocalization

We used the FUSION[31] pipeline to perform TWAS on the normalized cell-type-specific expression estimates and normalized bulk expression measures; we residualized each expression matrix by its first 50 principal components to account for variation due to technical (non-biological) factors. Imputed SNPs were restricted to those that overlap with the 1000 Genomes LD reference panel, providing 272,652 SNPs on which the analysis could be performed. A window of 500 kb upstream and 500 kb downstream of the lead SNP for each eQTL was used as the *cis* region to be tested. Gene-trait pairs were selected on the basis of the best-performing model after 5-fold cross-validation, including for best unbiased linear predictor (BLUP), elastic net (ENET), least absolute shrinkage and selection operator (LASSO), and just using the top SNP.

We tested for colocalization of GWAS and eQTLs by using the –coloc flag within the FUSION/TWAS pipeline. Colocalization is only performed in those gene-trait associations with $p < 0.05$. In each cell type, we tested eGenes with a significant association between expression and SNP (Tables 3 and 4). We report SNPs with a colocalization posterior probability (PP4) > 0.80.

## Cell-type-specific regressions using estimated cell-type proportions and gene expression

We built logistic regression models to evaluate the effect of cell-type proportion on case or control status, as well as lithium-use status within only the BP individuals. These models included the proportion of one cell type at a time, along with covariates including age, sex, RNA concentration, and RNA integrity number (RIN) as predictors. In testing the differences in cell-type proportions between different binary outcomes, we used the glm() function in R with family = binomial.

## Electronic-medical-record validation cohort

ATLAS is an opt-in biobank that enrolls individuals when they visit UCLA for a blood draw. ATLAS is a diverse biobank that includes individuals who come from a variety of genetic ancestries and live across the greater Los Angeles region.[32] Registered ATLAS researchers can access deidentified electronic-health-record data for individuals; these data consist of outpatient and inpatient encounters, including information on diagnoses, procedure orders, laboratory orders, and prescription orders. As of 2022, there were approximately 50,000 participants enrolled in ATLAS. A complete description of the ATLAS project and data is available in Johnson et al. (2022).[33] Recruitment and sample collection for the UCLA ATLAS dataset is approved by the UCLA IRB #17–001013. Informed consent was obtained from all participants.

Individuals with bipolar disorder were identified in ATLAS according to the diagnosis table. A person was defined as having a bipolar phenotype if the individual had at least one diagnosis of any of the ICD 10 codes included in the bipolar Phecode Map 1.2.[34] Neutrophil counts (measured as $10^3$ counts/μL) were determined from test results for complete-blood-count laboratory orders. We restricted this analysis to those individuals with self-reported European ancestry. To prevent severe outliers from biasing results, we removed test results with a neutrophil count greater than 2 standard deviations from the median count value in all bipolar individuals. Lithium prescription orders were found via a query of the prescription-order table for medications of any dose or format that was classified as psychiatric medication and had the generic name lithium.

Neutrophil-count data for individuals with a bipolar Phecode were separated into three categories: tests administered before the individual was prescribed lithium, tests administered after the first lithium prescription order, and tests for individuals without a lithium prescription order. Because many individuals had multiple complete blood-count orders, the median neutrophil count per individual per category was calculated. Median neutrophil counts were compared between bipolar individuals after their first lithium prescription and bipolar individuals without a lithium prescription via a logistic regression (implemented in R). Maximum age and sex were used as covariates. For the subset of individuals who had complete blood-count tests taken before and after a lithium prescription order, we used a paired Wilcoxon rank test, implemented in R with the wilcox.test (paired = TRUE) command, to increase power.

### Interaction model

To test whether there exists an interaction between SNP and lithium usage, we included an interaction component in the regression model, as such:

$$y = \beta 1 * X + \beta 2 * l + \beta 3 * (X * l) + covariates$$

where $X$ refers to the genotype at a particular SNP and $l$ refers to lithium use. Covariates include the first 50 expression PCs, age, sex, RNA concentration, and RNA integrity values.

### Differential expression analysis

We used the limma eBayes function[35] with trend = true to conduct differential expression tests in the bulk dataset. We include only those genes with at least 1 TPM in at least 436 individuals (about 25% of the total 1,730 individuals included in the analysis), leaving 17,194 genes to be tested. We then log2-transformed this matrix and computed the first 50 expression principal components to be included as covariates. In the lithium user vs. non-user analysis, we included only diagnosed individuals to avoid confounding effects caused by disease status, whereas in the case-control analysis, all individuals diagnosed with BP or SCZ were included as cases, and non-affected individuals were included as controls.

For the cell-type-specific differential expression analysis, we used the bmind_de() function as included in the bMIND software package. To keep the methods comparable to the bulk analysis, we also used the log2-transformed expression measures as inputs along with the first 50 expression PCs as covariates.

## Results

### Computationally derived cell-type estimates are reliable

The graphical abstract provides an overview of the pipeline used in this study to generate putative cell-type-specific eQTLs. To estimate cell-type gene expression in whole blood, we analyzed bulk-blood RNA-seq (n = 1,730) by using computational deconvolution tools. First, we estimated cell-type proportions by using the LM22 signature matrix and CIBERSORTx (Figure 1A and Table 1). We found that these proportion estimates are consistent with standard white blood cell reference ranges,[36] for which generally neutrophils have the highest abundance, lymphocytes (including T cells, B cells, and natural killer [NK] cells combined) the second highest abundance, and monocytes the lowest abundance. However, we note that blood cell-type proportions vary across individuals depending on numerous factors, such as medication use, current illness, and age.[37] We confirmed that the proportions estimated via CIBERSORTx are consistent with the complete blood-count measures taken in the clinic for a subset (n = 143) of individuals in our dataset (Figure S1). We observed a Pearson correlation ($R^2$) of- 0.85 for lymphocytes, 0.48 for monocytes, and 0.76 for cell-type proportions estimated in neutrophils by using CIBERSORTx and proportions measured in the clinic. These results suggest that the computationally estimated proportions are reliable.

Next, we used these proportion estimates and bMIND expression deconvolution (see subjects and methods) to estimate cell-type expression. Consistent with biological expectations, we found that correlation of estimated expression, measured by $R^2$ of the mean expression across samples per gene, between different cell types is high, as all cell types are derived from the same tissue (Figure 1B). Next, we investigated whether computationally estimated cell-type expression could successfully detect differences in expression between different cell types, despite a high correlation structure between different cell types. Principal-component analysis confirmed that the major sources of variation in the dataset are attributable to differences in cell-type expression (Figure S2). These results suggest that using large cohorts of bulk RNA-seq in blood, along with computational deconvolution tools, can successfully detect differences in expression on the basis of on cell-type composition.

Finally, we contrasted computationally derived cell-type gene-expression estimates with single-cell RNA-seq (scRNA-seq) data[38,39] (available online at https://dice-database.org/downloads#expression_download and https://github.com/eQTL-Catalogue/eQTL-Catalogue-resources/blob/master/tabix/tabix_ftp_paths.tsv). We compared median TPM (transcripts per million) estimates across six cell types and found a high correlation between the reference single-cell expression and computationally derived expression; $R^2$ ranged from 0.61 in naïve B cells to 0.84 in naïve CD4$^+$ T cells. (Table S3; Figure S3). To further check how well computationally estimated expression compares to expression derived from scRNA-seq, we correlated expression estimates between the two reference scRNA-seq datasets in monocytes, the one cell type with data available in both reference datasets. We found that the median TPM of more than 17,000 genes present in both datasets have an $R^2$ of 0.91, higher yet comparable to the $R^2$ observed when one compares computationally estimated expression with scRNA-seq.

### Cell-type eQTL analysis reveals more refined biological signal than bulk eQTL

Next, we performed eQTL analyses on the resulting cell type expression estimates to find evidence of genetic regulation of cell type expression. We restricted to the eight cell types with average proportion >2% including: naïve B Cells, memory B Cells, CD4 naïve T Cells, CD4 memory T cells, natural killer cells, monocytes, and neutrophils. We conducted local-eQTL mapping with a 1 Mb window by using QTLtools (see subjects and methods) to identify between 2,875 and 4,629 eGenes with a significant association at an FDR correction level of 5% across the eight different cell types (Figure 2A). In total, we identified 5,752 eGenes with a significant association in at least one of the eight main cell types (Table 1). We show that there exists a range in the condordances of effect sizes for eGenes found in both the individual cell-type analyses and the bulk eQTL analysis (Figures 2B and 2C). We computed Storey's $\pi_1$, a measure of replication rate, between the computationally derived eQTL and the bulk

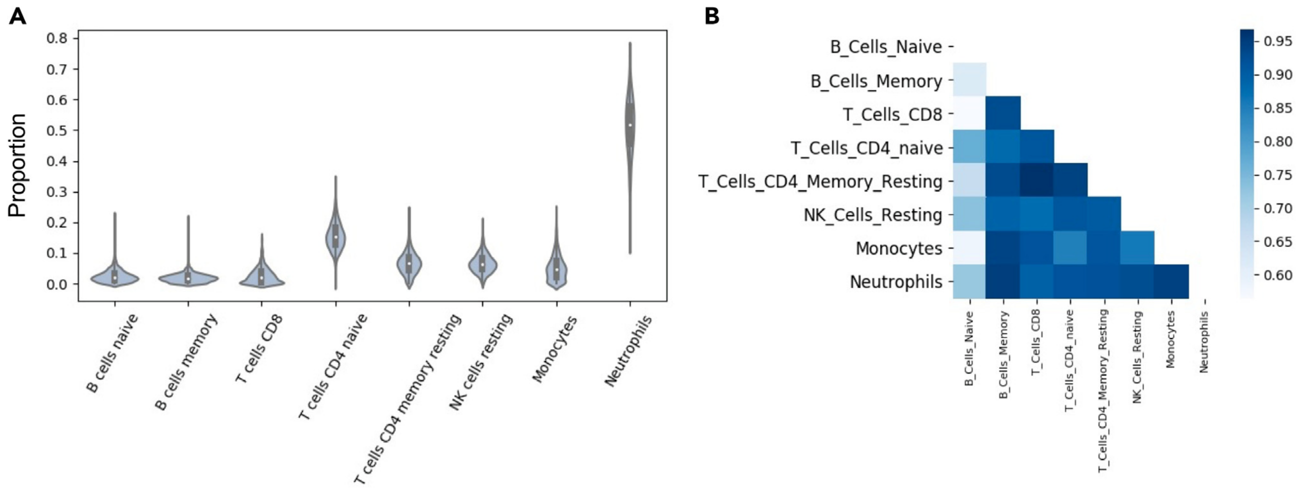


**Figure 1. Cell-type-specific expression from computational deconvolution methods**
(A) Cell-type-proportion predictions from CIBERSORTx. A violin plot showing the range of estimated cell-type proportions for all 1,730 individuals in each of the eight major cell types.
(B) $R^2$ of expression between each cell type. A heatmap of correlations (measured by $R^2$ of mean expression across samples) between the eight main cell types captured by CIBERSORTx.

eQTL and found rates at least 70% or greater, suggesting that the majority of eQTLs are replicated within each cell type. This confirms findings from previous studies showing a strong shared genetic effect on gene expression across cell types. We observed that most eGenes are detected as significant in either just one or all eight cell types (Figure S4).

Additionally, we found evidence of cell type "opposite-effect" eQTLs, where an SNP in a given cell type shows an association with the same eGene as that detected through bulk RNA-seq, but in the opposite direction. One such example is rs150248941, the eQTL for *FCGR3B* (Fc fragment of IgG receptor IIIb); whereas the bulk eQTL had an effect size of $-1.3$, the effect size in neutrophils and T cell types ranged between 0.49 and 0.86, and the remaining cell types had large negative effects. Similarly, rs60323161, the eQTL for *MACF1* (microtubule actin cross-linking factor 1) had effect sizes between $-1.1$ and $-0.15$ for the T cell types, versus effect sizes ranging between 0.21 and 0.28 for the bulk and remaining immune cell types. *MACF1* is known to be involved in neurite growth during brain development and has previously been linked to schizophrenia.[40] These examples are especially interesting because they support the idea that gene expression at the cell-type level can uncover nuances of biological mechanisms that go undetected when only bulk-level analyses are used. Similar effects have been observed in other studies involving both single-cell RNA-seq[41] and deconvoluted bulk RNA-seq.[42]

To further validate these cell-type eQTLs, we compared the results of this analysis with results from eQTL analysis by using single-cell RNA-seq from the eQTLCatalogue.[38,39,43,44] (uniformly processed summary statistics[44] are available at https://www.ebi.ac.uk/eqtl/Data_access/). We restricted the list of computationally derived eGenes included in the condordance analysis to the protein-coding genes. Generally, we found that the two approaches to cell-type eQTL mapping show strong concordance. For example, in neutrophils, we found that 2,921 out of the 4,629 genes (63%) with a significant association according to the computational deconvolution approach also had a significant association according to the single-cell RNA-seq. Among these eGenes, and comparing the association with the same leading SNP in both of these datasets (Figure 2D), we observed a correlation ($R^2$) of 0.66 between their effect sizes. Similar effect-size correlations for T cells CD4, B cells, and monocytes are shown in Figure S5. Similarly, we computed Storey's $\pi_1$ between computationally derived eGene effect sizes and these reference sc-RNAseq eGene effect sizes and found rates >99% for all comparisons. We also tested the replication with eGene data from the recent OneK1K[45] study (available online at https://onek1k.org/) as a further comparison for monocytes; memory and naïve B cells; $CD4^+$ and $CD8^+$ T cells; and NK cells and again found rates >99% for all comparisons. This suggests that the computational deconvolution approach to large-scale bulk RNA-seq projects can be used for obtaining accurate cell-type eQTL estimates.

## Integration of cell-type-specific eQTLs with brain- and blood-trait GWASs

For every gene with a significant eQTL, we used FUSION[31] to estimate the gene expression heritability across each of the contexts, or the proportion of variance in gene expression explained by variance in genetics. Only those genes with significant heritability after 5-fold cross-validation per each context were retained for further analysis. Table 2 provides the summarized statistics of the significantly heritable genes and the gene with highest estimated SNP heritability per cell type. An advantage of investigating eQTLs

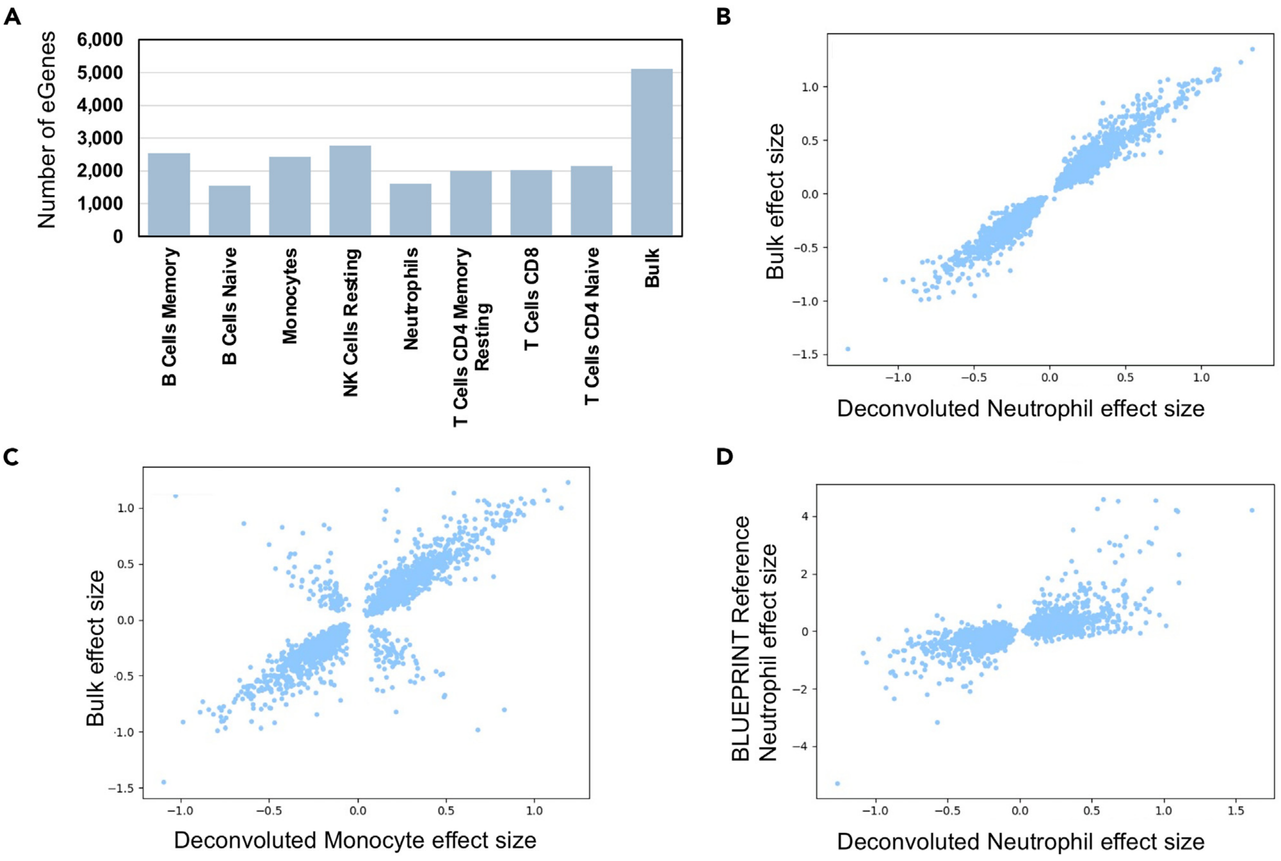


**Figure 2. eGenes per cell type and correlations between effect sizes**
(A) Number of eGenes with a significant association identified for the eight major cell types detected by CIBERSORTx; an FDR cutoff of 0.05 was used.
(B) Comparison of effect size between shared *cis* associations with neutrophils. Restricting eGenes to those with a significant association in both the bulk eQTL analysis and neutrophil eQTL analysis, we compared the estimated effect sizes of the most significant eQTL associations.
(C) Comparison of effect size between shared *cis* associations with monocytes. Restricting eGenes to those with a significant association in both the bulk eQTL analysis and the monocyte eQTL analysis, we compared the estimated effect sizes of the most significant eQTL associations.
(D) Comparison, using reference single-cell RNA-seq, of effect sizes between shared *cis* associations. Restricting eGenes to those with a significant association in both the BLUEPRINT reference neutrophil eQTL analysis and our neutrophil eQTL analysis, we compared the estimated effect sizes of the most significant eQTL associations.

at the cell-type level is that it provides a more precise view of biological mechanisms driving the association between gene expression and phenotype. In order to investigate whether there exist variants that drive both the expression of genes in a specific cell type and a GWAS trait, we conducted transcriptome-wide association study (TWAS)[31] and colocalization[46] analyses by using the significant cell-type-eQTLs from the eight main cell types previously mentioned, along with GWASs of several neuropsychiatric and blood-based phenotypes. Figure 3A provides an overview of the overlap across the contexts, both for brain-related and blood-based traits.

GWASs for neuropsychiatric traits tested include: BP,[50] SCZ,[51] major depressive disorder (MDD),[52] alcohol dependence,[53] cannabis-use disorder,[54] migraines,[55] insomnia,[56] attention-deficit/hyperactivity disorder (ADHD),[57] and Alzheimer disease.[58] In total there were 710 eGenes found to be associated only in the bulk and in no other cell type (or in other words, the set difference between the bulk eGenes and the union of all cell-type-specific eGenes) and 168 eGenes found to be associated in one or more cell types and not in the bulk (Table 3). Regarding colocalization, in total there were 68 eGenes found to have colocalized SNPs between expression and traits only in the bulk and in no other cell type, and 50 eGenes found only in one or more cell types and not in the bulk (Table 3).

Of the 50 eGenes found to have a colocalization posterior probability with the same variant impacting both gene expression and the GWAS trait (PP4 $> 0.8$) in a cell type but not in the bulk, half have a higher median TPM across the GTEx v.8 brain tissue types than in GTEx whole blood. This suggests that these genes are relevant for brain functions despite being detected in immune-cell-type-specific expression estimates. An example of one such gene is *HTR6*, a serotonin receptor targeted by certain antidepressant and antipsychotic medication and found to be

Table 1. **Cell-type-proportion estimates from CIBERSORTx and number of eQTLs per cell type**

| Cell type | Mean cell-type-proportion estimate (s.d.) | Number of eGenes (FDR $< 0.05$) |
|---|---|---|
| Naive B cells | 0.025 (0.020) | 4,009 |
| Memory B cells | 0.020 (0.014) | 3,571 |
| CD8 T cells | 0.025 (0.025) | 2,875 |
| Naive CD4 T cells | 0.15 (0.042) | 3,082 |
| Memory resting CD4 T cells | 0.066 (0.034) | 3,284 |
| Resting NK Cells | 0.066 (0.029) | 3,858 |
| Monocytes | 0.050 (0.039) | 3,483 |
| Neutrophils | 0.51 (0.094) | 4,629 |
| Bulk (directly from RNA-seq) | 1.0 | 7,302 |

strongly associated and colocalized with BP in the most recent Psychiatric Genomics Consortium (PGC) study on bipolar disorder.[50] This study used brain-derived gene expression weights from the PsychENCODE project.[59] Conditioning on *HTR6* memory-B-cell-specific expression by using FUSION completely removed the significant GWAS signal at this locus, suggesting that the genetic factor driving gene expression also encompasses the BP association signal (Figure 3B). The same held true for other immune cell types in which *HTR6* was colocalized with BP; such cell types included naïve B cells and CD4 T cells. This demonstrates the utility of using cell-type deconvolution methods in large cohorts of an easily accessible tissue such as blood because it is able to capture gene-expression regulation relevant in brain cell types that otherwise are not detectable in bulk-blood eQTLs.

GWASs for blood-based traits tested include systemic lupus erythematosus[60] (an autoimmune disorder), mean corpuscular volume, mean corpuscular hemoglobin,[61] red blood cell width distribution, monocyte count, eosinophil count, lymphocyte count, platelet count, white blood cell count, and red blood cell count.[62] In total there were 1,765 eGenes found to have associations only in the bulk and in no other cell type, and there were 493 eGenes found only in one or more cell types and not in the bulk (Table 4). Regarding colocalization, in total there were 488 eGenes found only in the bulk and in no other cell type and 229 eGenes found only in one or more cell types and not in the bulk (Table 4).

Within the blood-based traits, we again found examples of opposite-sign effects in certain cell types when compared to the bulk. For example, when considering systemic lupus erythematosus (SLE) as a trait, we found that for *IRF5*, natural killer cells have a TWAS $Z$ score of $-10.7$, whereas the bulk has a score of $+3.91$, suggesting distinct mechanisms that are dependent on the cell-type context. IRF5 (interferon regulatory factor 5) is known to be implicated in SLE,[63,64] although the exact mechanism by which it is dysregulated in the context of disease remains unknown.

Table 2. **FUSION heritability results**

| Cell type | Number of significant genes | Min | Q1 | Median | Mean | Q3 | Max | Gene with max $h^2$ |
|---|---|---|---|---|---|---|---|---|
| **Bulk** | 5,113 | 0.0041 | 0.026 | 0.055 | 0.096 | 0.12 | 0.728 | *TRBV28* |
| **Memory B cells** | 2,541 | 0.0035 | 0.024 | 0.044 | 0.075 | 0.093 | 0.68 | *BTG1* |
| **Naive B cells** | 1,552 | 0.0056 | 0.024 | 0.045 | 0.078 | 0.095 | 0.579 | *PI16* |
| **Monocytes** | 2,431 | 0.0052 | 0.025 | 0.045 | 0.077 | 0.095 | 0.584 | *NSG1* |
| **Resting NK cells** | 2,763 | 0.0042 | 0.024 | 0.045 | 0.078 | 0.098 | 0.61 | *BCAT1* |
| **Neutrophils** | 1,605 | 0.0056 | 0.025 | 0.048 | 0.083 | 0.10 | 0.69 | *CAMKK2* |
| **Resting memory CD4 T cells** | 1,989 | 0.0057 | 0.026 | 0.047 | 0.080 | 0.099 | 0.63 | *SBF2* |
| **CD8 T cells** | 2,033 | 0.0057 | 0.024 | 0.042 | 0.069 | 0.081 | 0.63 | *FGFBP2* |
| **Naive CD4 T cells** | 2,147 | 0.0053 | 0.024 | 0.044 | 0.075 | 0.092 | 0.56 | *CROT* |

Number of significant genes refers to the number of genes that remain significantly ($p < 0.05$) heritable after five-fold cross-validation. Q1 = first interquartile, Q3 = third interquartile. Overall, the bulk data show higher heritability estimates across each of the statistics. Of note is that every gene listed is distinct for each context; this includes genes that are relevant to neuronal function, such as *NSG1* (neuronal vesicle trafficking associated),[47] *CAMKK2* (calcium-dependent kinase, involved in neuronal differentiation and synapse formation),[48] and *BTG1* (B-cell translocation gene 1, found to be involved in neural stem cell renewal).[49].

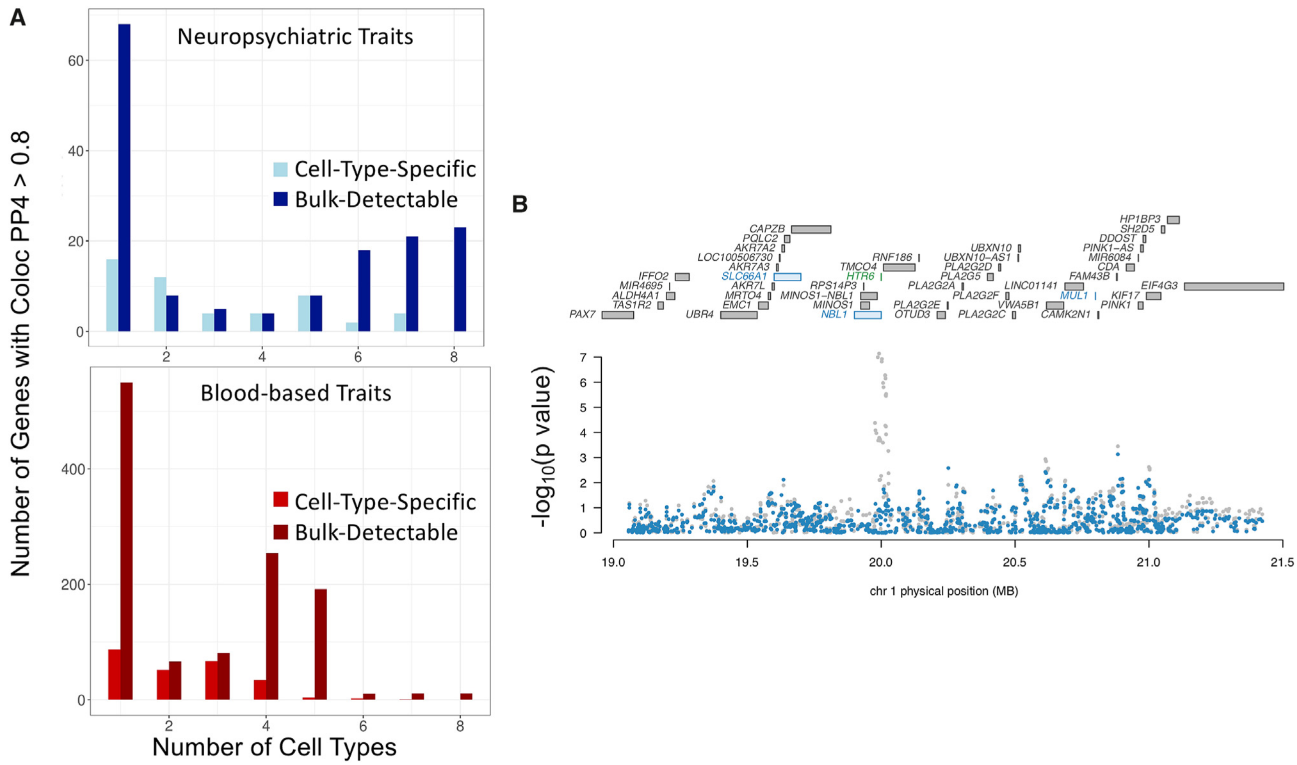


**Figure 3. Colocalization and enrichment analyses of cell-type-specific eQTLs**
(A) (Top) Number of genes with coloc PP4 > 0.8 across contexts in neuropsychiatric traits. (Bottom) Number of genes with coloc PP4 > 0.8 across contexts in blood-based traits.
(B) Conditional analysis of *HTR6* expression in memory B cells. All genes in the locus are included in the top panel; marginally TWAS-associated genes are highlighted in blue, and those jointly significant (*HTR6*) are in green. The bottom panel includes a Manhattan plot of the GWAS data before (gray) and after (blue) conditioning on the imputed expression of *HTR6* in memory B cells. Imputed expression of *HTR6*, including 238 *cis*-SNPs in a LASSO regression model, was obtained. Figure generated by FUSION.post_process.R script.

See Tables S4, S5, S6, S7, S8, S9, S10, S11, and S12 to view all FUSION TWAS and colocalization results.

## Lithium-dependent genetic regulation of gene expression

Given the large number of BP probands in our study sample, we were interested to see whether there were BP-specific effects that could be observed via cell-type-deconvoluted expression. Because lithium is the most commonly used drug for treating these individuals and because it has also been established that lithium use has an effect on the blood transcriptome,[65,66] we hypothesized that lithium-dependent genetic regulation of the blood transcriptome might exist. Among the 1,045 bipolar individuals in this cohort, 709 were taking lithium at the time of blood draw ("lithium user") and 336 were not ("lithium non-user").

We set out to replicate our earlier findings from Krebs et al.[66] in a larger independent cohort. When stratifying by cases versus controls (with all BP and SCZ individuals included as cases), we found significant differences in the cell-type proportion for CD4 T cells ($p = 1.8 \times 10^{-7}$, higher in controls), natural killer resting cells ($p = 1.2 \times 10^{-7}$, higher in controls), and neutrophils ($p = 2.3 \times 10^{-8}$, higher in cases). Next, considering only the individuals with BP, we stratified those who use lithium versus those who do not and found significant differences in cell-type proportion for CD4 naïve T cells ($p = 8 \times 10^{-4}$, higher in non-users), CD4 memory T cells ($p = 4 \times 10^{-4}$, higher in non-users), natural killer resting cells ($p = 3 \times 10^{-4}$, higher in non-users), and neutrophils ($p = 1.5 \times 10^{-9}$, higher in users). However, when we only included lithium non-users within the BP individuals and compared those against the controls, we found no significant differences in proportion for any of the cell types. See Figure S6 for example plots of all three tests using neutrophils. This replicated our previous findings in a larger but independent sample recruited as part of the same cohort,[66] suggesting that lithium use, rather than disease status itself, by the BP individuals drives these differences in cell-type proportion.

We further validated the effect of lithium use on blood cell types in a separately ascertained cohort of individuals who had electronic health data from the University of California, Los Angeles ATLAS Community Health Initiative.[33,67] Specifically, we included self-reported European individuals who had a Phecode for bipolar disorder and also had laboratory test orders for complete blood counts, and we noted whether they had a prescription order for lithium (n = 1,302 with lithium, n = 6,208 without). In comparing the neutrophil count between

**Table 3. TWAS and colocalization with neuropsychiatric traits**

| Cell type | Significant eGenes | Number of significant TWAS genes, shared | Number of significant TWAS genes, unique | Number of genes with coloc PP4 > 0.8, shared | Number of genes with coloc PP4 > 0.8, unique |
|---|---|---|---|---|---|
| **Naïve B cells** | 4,009 | 90 | 43 | 43 | 13 |
| **Memory B cells** | 3,571 | 142 | 58 | 62 | 25 |
| **CD8 T cells** | 2,875 | 108 | 50 | 50 | 15 |
| **Naïve CD4 T cells** | 3,082 | 120 | 46 | 56 | 19 |
| **Resting memory CD4 T cells** | 3,082 | 115 | 43 | 55 | 22 |
| **Resting NK cells** | 3,858 | 156 | 72 | 73 | 21 |
| **Monocytes** | 3,483 | 126 | 52 | 62 | 24 |
| **Neutrophils** | 4,629 | 76 | 35 | 35 | 9 |
| **Bulk** | 7,302 | 906 | - | 155 | - |

Shared refers to the number of significant (FDR $< 0.05$) genes that are in common with the bulk TWAS-significant gene set, whereas unique refers to those that are not present in the bulk TWAS-significant gene set.

bipolar individuals who had never been prescribed lithium (or before they were prescribed lithium) and those who had a prescription order for lithium, we found that there was a significant (logistic regression $p = 2.09 \times 10^{-7}$) elevation of neutrophils in individuals with a prescription for lithium (Figure S7). Furthermore, for a subset of bipolar individuals within the ATLAS dataset, we also have records for neutrophil counts both before and after the individual was prescribed lithium. Using a Wilcoxon-signed rank test with continuity correction, we found a significant difference between the neutrophil counts between the two groups ($p = 0.0228$) when we included individuals of any ancestry ($n = 376$), although when we restricted individuals to only European individuals ($n = 229$), the significant difference was lost ($p = 0.2$) (Figure S7). The replication of this finding in this large external dataset provides further evidence to suggest that cell-type proportion is impacted by lithium usage, although the implications of this are yet to be understood.

To investigate lithium-dependent genetic regulation, we performed an interaction model eQTL scan between lithium users and nonusers and tested whether there exist SNPs whose cell type or cell-type-specific expression regulation is dependent on the presence of lithium. To do this, we included an interaction term for the genotypes and lithium status in the regression model (see subjects and methods). Using bulk expression, we only identified one gene with such an association (FDR p value $< 0.10$). With cell-type expression derived from bMIND, we identified as many as 34 such eGenes (in monocytes) and a total of 82 unique examples of genes (Li-eGenes) that show differential regulation of cell-type expression (Table S13), in comparison to just one gene that shows differential regulation of bulk expression. We found that 97 of the eGenes that have significant differential lithium regulation exhibit opposite effect sizes between the lithium user and nonuser groups at the cell-type level. The remaining 13 Li-eGenes show same direction of effect sizes between the lithium

**Table 4. TWAS and colocalization with blood-based traits**

| Cell type | Significant eGenes | Number of significant TWAS genes, shared | Number of significant TWAS genes, unique | Number of genes with coloc PP4 > 0.8, shared | Number of genes with coloc PP4 > 0.8, unique |
|---|---|---|---|---|---|
| **Naïve B cells** | 4,009 | 922 | 164 | 289 | 78 |
| **Memory B cells** | 3,571 | 1,582 | 207 | 511 | 106 |
| **CD8 T cells** | 2,875 | 1,276 | 168 | 414 | 93 |
| **Naïve CD4 T cells** | 3,082 | 1,349 | 183 | 445 | 88 |
| **Resting memory CD4 T cells** | 3,082 | 1,257 | 150 | 419 | 80 |
| **Resting NK cells** | 3,858 | 1,712 | 254 | 557 | 119 |
| **Monocytes** | 3,483 | 1,484 | 212 | 484 | 113 |
| **Neutrophils** | 4,629 | 969 | 159 | 331 | 60 |
| **Bulk** | 7,302 | 3,893 | / | 1,175 | / |

Shared refers to the number of significant (FDR $<0.05$) genes that are in common with the bulk TWAS-significant gene set, whereas unique refers to those that are not present in the bulk TWAS-significant gene set.

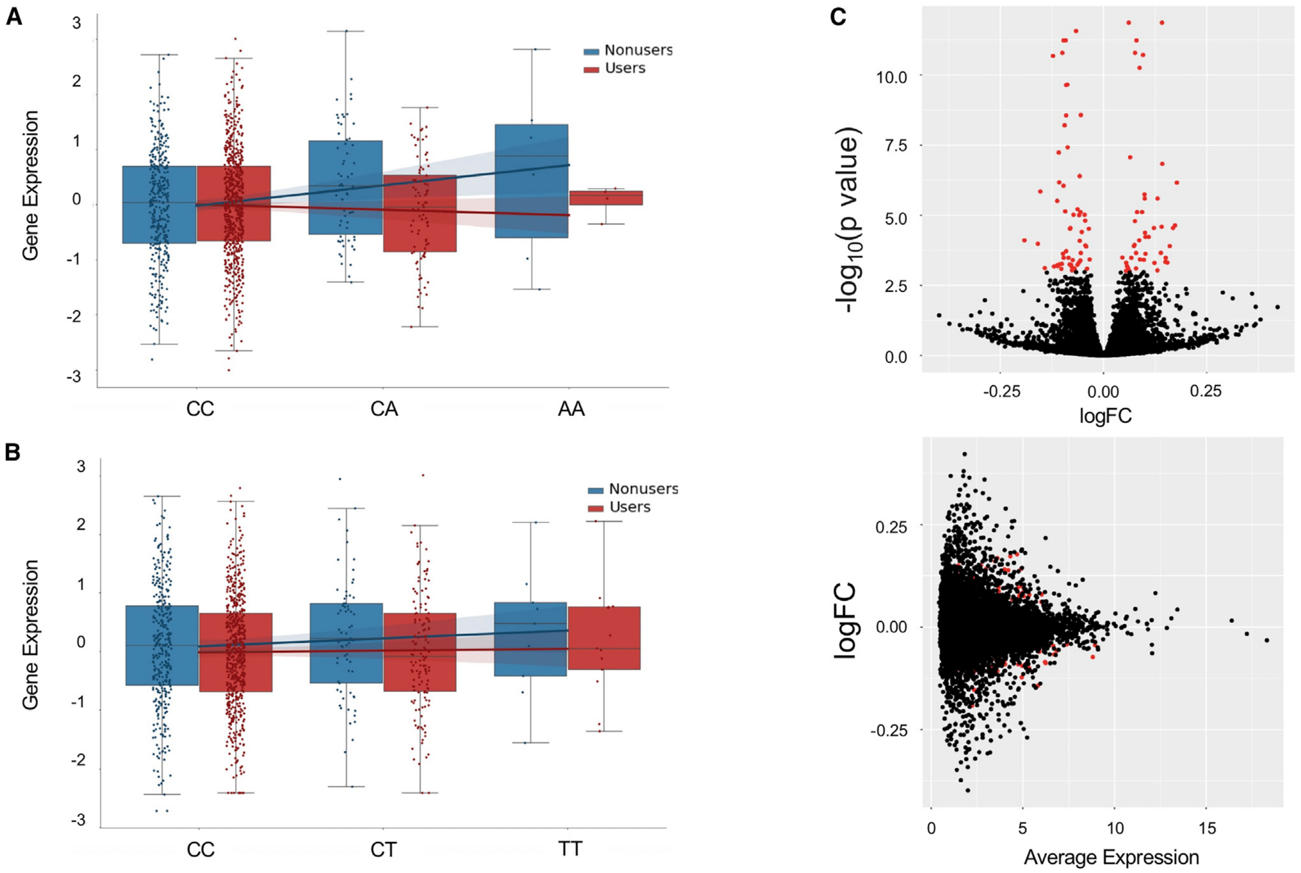


**Figure 4. Lithium user vs. non-user analyses**
(A) Boxplots showing the normalized expression of *KITLG* (Ensembl: ENSG00000049130) in naïve B cells, stratified by dosage of SNP rs73207047 in lithium users versus nonusers. Median values are shown as a line in the box; whiskers of boxplots are 1.5 times the interquartile range.
(B) Boxplots showing the normalized expression of *TNFRSF11A* (Ensembl: ENSG00000141655) in monocytes, stratified by dosage of SNP rs79143095 in lithium users versus nonusers. Median values are shown as a line in the box; whiskers of boxplots are 1.5 times the interquartile range.
(C) Differential gene expression results for lithium users vs. lithium non-users. (Top) Volcano plot that highlights differentially expressed genes (FDR $< 0.05$) in red ($n = 100$ total differentially expressed genes). (Bottom) Average expression of each gene vs. the log fold-change (logFC) of each gene; differentially expressed genes are highlighted in red.

user and nonuser groups, but with significantly different magnitudes (Table S14 for summarized results). For example, in naïve B cells, *KITLG* (ENSG00000049130) shows opposite effect eQTLs on the basis of rs73207047 (Figure 4A), whereas in monocytes we see that *TNFRSF11A* (ENSG00000141655) shows differential effect size, in the same direction, on the basis of rs79143095 (Figure 4B). Due to the large number of samples used in this analysis, we are powered to detect small differences such as these.

In order to directly measure expression differences between lithium users and nonusers, we conducted a differential expression analysis test by using limma[68] initially in the bulk dataset (see subjects and methods). Comparing the two groups, we tested 17,194 genes from bulk-expression measures. We found 100 genes with evidence of differential expression in the bulk (FDR $< 0.05$); log fold-changes of the significant genes ranged from $-0.191$ to 0.177, suggesting the low impact of lithium on differential expression (Figure 4C). Out of the 100 differentially expressed genes found here, 33 were previously reported in Krebs et al.;[66] this overlap is significant according to Fisher's exact test (OR $= 6.43$, $p = 4.74 \times 10^{-14}$). Overlapping genes include *FBXL2*, a gene highly expressed in the brain and involved in neuronal signaling, and *CNTNAP3*, which mediates interactions between neurons and glial cells. See Table S15 for full lithium differential expression results.

Although previous studies have not found substantial evidence of differential expression in the blood transcriptome between individuals with BP or SCZ and controls,[66,69] we were interested in investigating this within our own cohort given the uniquely large sample size. Using the bulk RNA-seq and the same 17,194 genes selected in the lithium-user differential expression analysis, we found 64 genes with FDR $< 0.05$; of these, nine genes overlapped with the significant genes found in the lithium analysis. Log fold-changes of the significant genes ranged only from $-0.126$ to 0.104, suggesting that if these genes are truly a result of disease status, the differences are minimal

(Figure S8). See Table S15 for full case/control differential expression results.

For the cell-type-specific differential expression analyses, we leveraged the differential-expression function available through the bMIND software. In the case-control analysis, we found four differentially expressed genes in neutrophils; these genes included *TSPAN2* and *CFAP45*, both of which were reported in the Krebs et al. lithium differential expression study.[66] We found 24 differentially expressed genes in memory B cells and 21 in naïve B cells (and 18 differentially expressed genes in common between the two B cell types). Interestingly, when conducting the lithium user versus non-user analysis, we did not find any differentially expressed genes in any cell type. Although this could be a result of the smaller sample set used in the lithium analysis than in the case-control analysis, it also might reflect the fact that the effects of lithium are only found at the bulk level because of its impact on cell-type composition, rather than reflecting changes in gene expression within individual cell types. To test whether bulk expression data can still detect differentially expressed genes even with adjustments in cell-type proportions, we tested the inclusion of the cell-type proportions as covariates (in addition to the 50 expression PCs) in the bulk lithium differential-expression test. We found 94 differentially expressed genes, 82 of which were significant in the original version of the analysis (without cell-type proportions as covariates), a significant overlap (Fisher's $p < 2 \times 10^{-16}$), suggesting that adjusting for cell-type proportions still allows for the detection of differentially expressed genes in bulk data. See Table S15 for q values of all cell-type-specific differential-expression results.

## Discussion

We show that cell-type deconvolution of bulk-blood RNA-seq provides insights not only for immune-relevant biology but also for neuropsychiatric disease biology. Although bulk eQTLs tend to provide a greater number of associations overall, we find that cell-type-specific eQTLs provide unique associations not otherwise detectable in the bulk. Many of these unique cell-type associations have high expression in brain tissue types and harbor several example genes that have been previously implicated in BP TWASs[50] using brain tissue. This demonstrates that large cohorts of an easily accessible tissue such as blood are useful for deciphering biology for brain-related phenotypes when cell-type deconvolution is applied.

An important caveat, however, is that the associations with brain-related traits found in this study are most likely to be shared genetic mechanisms between blood cell types and brain cell types, rather than being blood-cell-type-specific biology. Relatedly, because there are most likely many brain-specific gene-expression regulation mechanisms relevant to disease biology, there are limitations on how much information is available from blood. Mechanistic insights lag behind particularly for brain-related traits because of the inaccessibility of living brain tissue. Postmortem gene expression has been shown to be very different from gene expression in living brain tissue,[70] and thus there is a need for accessible tissue or biofluid samples from living donors. Although gene expression is not highly correlated between different tissue types, the *cis* genetic effects are highly correlated,[17] suggesting the potential to still gain useful information from more accessible procedures such as blood draw, although it is not the full picture. The advancement of procedures akin to those described in Liharska et al.[70] allows for the safe biopsy of living brain tissue during neurosurgery, paving the way for genomic studies from these understudied samples.

Considering the BP TWAS results alone, we found 82 total eGenes with an opposite direction of effect in a cell type than in the bulk eQTL analysis (an eGene was defined as having an opposite direction of effect if there was an opposite-sign TWAS $Z$ score for the same gene and the same trait). For example, we found 63 eGenes, significantly associated with BP, that have an opposite direction of effect in CD8 T cells than in bulk expression. *ARID5A*, a gene implicated in the most recent TWAS on PGC bipolar disorder,[50] is one example of these genes. In the bulk expression, the TWAS $Z$ score of *ARID5A* and bipolar disorder is −4.99 (TWAS $Z$ score −5.32 in PGC BP study), whereas in CD8 T cells it is +6.02. With PP4 > 0.8 in the CD8 T cell test, this gene was also found to be colocalized, although it does not pass the colocalization threshold in the bulk test or PGC3 BP test. The same is true for *ARID5A* in CD4 memory resting T cells (TWAS $Z$ score +6.56). Similarly, the methyltransferase gene *WDR82* in CD4 naïve T cells has a positive association (TWAS $Z$ score +3.72) with BP, whereas the bulk expression has a negative association (TWAS $Z$ score −3.98) at the same locus (TWAS $Z$ score −6.75 in PGC BP study). These opposite directions of effect dependent on cell type have been found previously, both in blood[4] and brain[4,28] contexts. Similar to our finding that some genes with significant associations with traits in cell-type-specific contexts are not detectable via bulk expression, these previous studies[4,28] have also found such examples.

Additional BP-associated genes include *RILPL2*, found to be colocalized in the context of memory B cells, monocytes, natural-killer resting cells, and CD8 T cells, but not in the bulk. This gene is highly expressed in whole blood in adults (median TPM 27.42 in GTEx) but is also crucial for dendritic-spine morphogenesis in developing neurons.[71] Similarly, *CAMKK2* (calcium/calmodulin-dependent protein kinase kinase 2), a gene found to be colocalized in the context of monocytes, neutrophils, and CD4 T cells, is highly expressed both in whole blood and in brain tissues (particularly

cerebellar hemisphere and cerebellum, according to GTEx). Although *CAMKK2* has not been implicated in a BP TWAS, the large PGC GWAS points toward calcium-channel signaling as a potential therapeutic target for BP,[50] and indeed a loss-of-function mutation in this gene has been previously linked to BP status.[72] We consider these to be potential BP-relevant genes that are interesting candidates for experimental validation.

We replicated previous findings that immune-cell-type composition is impacted by lithium use rather than BP status. We also replicated several previously reported genes that are differentially expressed in whole blood in response to lithium, in addition to reporting additional lithium-response genes. Although lithium has been prescribed as a mood stabilizer for decades, its precise mechanism of action is still unclear.[73] Lithium has been shown to increase the activity of the transcription factor CREB (cAMP response element-binding protein),[74] a protein involved in neuronal plasticity.[75] Here, we found that *ATF4*, which encodes for CREB-2 and is an eGene in all cell types and the bulk, has opposite directions of effect in T cell types than in the other immune cell types or the bulk. We found a similar pattern for the *AKT1* (Rho-family-alpha serine/threonine-protein kinase) eGene. AKT1 levels in brain tissue have been previously associated with both schizophrenia and bipolar disorder, and although genetic associations exist,[76] they do not pass genome-wide multiple-testing correction.

Although we found promising lines of evidence that immune-cell-type-specific expression is useful for discovering candidate brain-relevant genes, there are several limitations to our study. Firstly, although our cohort had an ample number of bipolar samples, the number of SCZ samples was much lower and was thus underpowered for a diagnosis-specific analysis. Furthermore, we only tested SNP-gene pairs in *cis*, whereas *trans* eQTLs are known to be more context-specific,[77] so we missed distal associations that are potentially biologically relevant to the phenotypes of interest. Using computationally derived expression estimates creates a greater possibility for spurious associations that are not related to biology, dependent on the specific method of decomposition or deconvolution chosen. Also, by using low-coverage RNA-seq (average 5.9 million mapped reads per sample), we might have missed important eGenes that are not as highly expressed in blood. Finally, our study consists of all European-ancestry individuals, but to gain a more comprehensive and inclusive understanding of the biology between immune cell types and psychiatric conditions, in addition to better fine-mapping these eQTLs, future work will need to analyze many more samples of diverse ancestries.

Collectively, all of this suggests that although bulk whole-blood gene expression provides a greater number of significant findings overall, cell-type-specific expression allows us to observe additional biological mechanisms that are not possible to capture with gene-expression measures from the bulk alone.

## Data and code availability

The accession number for the RNA-seq data and corresponding genotypes resported in this paper is dbGAP: phs002856.v1.

## Supplemental information

Supplemental information can be found online at https://doi.org/10.1016/j.ajhg.2023.12.018.

## Acknowledgments

We are greatly appreciative of those individuals who donated the blood samples on which this study was based. We gratefully acknowledge the Institute for Precision Health, participating individuals from the UCLA ATLAS Precision Health Biobank, UCLA David Geffen School of Medicine, UCLA Clinical and Translational Science Institute, and UCLA Health. T.B. was supported by the National Institutes of Health (NIH) (grant no. 5T32HG002536-19). We also would like to thank Tanner Waters for his meaningful discussions on this work. This research was supported by the National Institute of Mental Health of the NIH under award no. 2R01MH115676-06, former number 5R01MH115676-05. The content is solely the responsibility of the authors and does not necessarily represent the official views of the National Institutes of Health. The graphical abstract was created with BioRender.

## Declaration of interests

Tommer Schwarz currently is employed at Cytoreason in Tel Aviv, Israel. The remaining authors declare no competing interests.

Received: June 5, 2023
Accepted: December 13, 2023
Published: February 1, 2024

## Web resources

FastQC, https://bioinformatics.babraham.ac.uk/projects/fastqc/

LM22 signature matrix (for noncommercial use only), https://cibersortx.stanford.edu/download.php

## References

1. Zeng, B., Bendl, J., Kosoy, R., Fullard, J.F., Hoffman, G.E., and Roussos, P. (2022). Multi-ancestry eQTL meta-analysis of human brain identifies candidate causal variants for brain-related traits. Nat. Genet. *54*, 161–169.
2. Zhang, J., and Zhao, H. (2023). eQTL studies: from bulk tissues to single cells. Journal of Genetics and Genomics *50*, 925–933.
3. Finucane, H.K., Bulik-Sullivan, B., Gusev, A., Trynka, G., Reshef, Y., Loh, P.-R., Anttila, V., Xu, H., Zang, C., Farh, K., et al. (2015). Partitioning heritability by functional annotation using genome-wide association summary statistics. Nat. Genet. *47*, 1228–1235.
4. Kim-Hellmuth, S., Aguet, F., Oliva, M., Muñoz-Aguirre, M., Kasela, S., Wucher, V., Castel, S.E., Hamel, A.R., Viñuela, A., Roberts, A.L., et al. (2020). Cell type-specific genetic regulation of gene expression across human tissues. Science *369*, eaaz8528.

5. Mandric, I., Schwarz, T., Majumdar, A., Hou, K., Briscoe, L., Perez, R., Subramaniam, M., Hafemeister, C., Satija, R., Ye, C.J., et al. (2020). Optimized design of single-cell RNA sequencing experiments for cell-type-specific eQTL analysis. Nat. Commun. *11*, 5504.
6. Schwarz, T., Boltz, T., Hou, K., Bot, M., Duan, C., Loohuis, L.O., Boks, M.P., Kahn, R.S., Ophoff, R.A., and Pasaniuc, B. (2022). Powerful eQTL mapping through low-coverage RNA sequencing. HGG Adv. *3*, 100103.
7. Avila Cobos, F., Alquicira-Hernandez, J., Powell, J.E., Mestdagh, P., and De Preter, K. (2020). Benchmarking of cell type deconvolution pipelines for transcriptomics data. Nat. Commun. *11*, 5650.
8. Jin, H., and Liu, Z. (2021). A benchmark for RNA-seq deconvolution analysis under dynamic testing environments. Genome Biol. *22*, 102.
9. Lu, A., Thompson, M., Grace Gordon, M., Dahl, A., Ye, C.J., Zaitlen, N., and Balliu, B. (2021). Fast and powerful statistical method for context-specific QTL mapping in multi-context genomic studies. Preprint at bioRxiv. https://doi.org/10.1101/2021.06.17.448889.
10. Aguirre-Gamboa, R., de Klein, N., di Tommaso, J., Claringbould, A., van der Wijst, M.G., de Vries, D., Brugge, H., Oelen, R., Võsa, U., Zorro, M.M., et al. (2020). Deconvolution of bulk blood eQTL effects into immune cell subpopulations. BMC Bioinf. *21*, 243–323.
11. Newman, A.M., Steen, C.B., Liu, C.L., Gentles, A.J., Chaudhuri, A.A., Scherer, F., Khodadoust, M.S., Esfahani, M.S., Luca, B.A., Steiner, D., et al. (2019). Determining cell type abundance and expression from bulk tissues with digital cytometry. Nat. Biotechnol. *37*, 773–782.
12. Wang, J., Roeder, K., and Devlin, B. (2021). Bayesian estimation of cell type-specific gene expression with prior derived from single-cell data. Genome Res. *31*, 1807–1818.
13. Chu, T., Wang, Z., Pe'er, D., and Danko, C.G. (2022). Cell type and gene expression deconvolution with BayesPrism enables Bayesian integrative analysis across bulk and single-cell RNA sequencing in oncology. Nat. Cancer *3*, 505–517.
14. Chen, Y., Wang, Y., Chen, Y., Cheng, Y., Wei, Y., Li, Y., Wang, J., Wei, Y., Chan, T.-F., and Li, Y. (2022). Deep autoencoder for interpretable tissue-adaptive deconvolution and cell-type-specific gene analysis. Nat. Commun. *13*, 6735.
15. Jew, B., Alvarez, M., Rahmani, E., Miao, Z., Ko, A., Garske, K.M., Sul, J.H., Pietiläinen, K.H., Pajukanta, P., and Halperin, E. (2020). Accurate estimation of cell composition in bulk expression through robust integration of single-cell information. Nat. Commun. *11*, 1971.
16. Khandaker, G.M., Dantzer, R., and Jones, P.B. (2017). Immunopsychiatry: important facts. Psychol. Med. *47*, 2229–2237.
17. Qi, T., Wu, Y., Zeng, J., Zhang, F., Xue, A., Jiang, L., Zhu, Z., Kemper, K., Yengo, L., Zheng, Z., et al. (2018). Identifying gene targets for brain-related traits using transcriptomic and methylomic data from blood. Nat. Commun. *9*, 2282.
18. Werner, M.C.F., Wirgenes, K.V., Shadrin, A., Lunding, S.H., Rødevand, L., Hjell, G., Ormerod, M.B.E.G., Haram, M., Agartz, I., Djurovic, S., et al. (2022). Immune marker levels in severe mental disorders: associations with polygenic risk scores of related mental phenotypes and psoriasis. Transl. Psychiatry *12*, 38.
19. (2015). Psychiatric genome-wide association study analyses implicate neuronal, immune and histone pathways. Nat. Neurosci. *18*, 199–209.
20. Korologou-Linden, R., Leyden, G.M., Relton, C.L., Richmond, R.C., and Richardson, T.G. (2021). Multi-omics analyses of cognitive traits and psychiatric disorders highlights brain-dependent mechanisms. Hum. Mol. Genet. *32*, 885–896.
21. Le Clerc, S., Lombardi, L., Baune, B.T., Amare, A.T., Schubert, K.O., Hou, L., Clark, S.R., Papiol, S., Cearns, M., Heilbronner, U., et al. (2021). HLA-DRB1 and HLA-DQB1 genetic diversity modulates response to lithium in bipolar affective disorders. Sci. Rep. *11*, 17823.
22. Andrews, S. (2010). FastQC: A Quality Control Tool for High Throughput Sequence Data.
23. Bray, N.L., Pimentel, H., Melsted, P., and Pachter, L. (2016). Near-optimal probabilistic RNA-seq quantification. Nat. Biotechnol. *34*, 525–527.
24. 1000 Genomes Project Consortium, Auton, A., Brooks, L.D., Durbin, R.M., Garrison, E.P., Kang, H.M., Korbel, J.O., Marchini, J.L., McCarthy, S., McVean, G.A., and Abecasis, G.R. (2015). A global reference for human genetic variation. Nature *526*, 68–74.
25. Lam, M., Awasthi, S., Watson, H.J., Goldstein, J., Panagiotaropoulou, G., Trubetskoy, V., Karlsson, R., Frei, O., Fan, C.-C., De Witte, W., et al. (2020). RICOPILI: Rapid Imputation for COnsortias PIpeLIne. Bioinformatics *36*, 930–933.
26. Aguet, F., Anand, S., Ardlie, K.G., Gabriel, S., Getz, G.A., Graubert, A., Hadley, K., Handsaker, R.E., Huang, K.H., Kashin, S., et al. (2020). The GTEx Consortium atlas of genetic regulatory effects across human tissues. Science *369*, 1318–1330.
27. Delaneau, O., Ongen, H., Brown, A.A., Fort, A., Panousis, N.I., and Dermitzakis, E.T. (2017). A complete tool set for molecular QTL discovery and analysis. Nat. Commun. *8*, 15452.
28. de Klein, N., Tsai, E.A., Vochteloo, M., Baird, D., Huang, Y., Chen, C.-Y., van Dam, S., Oelen, R., Deelen, P., Bakker, O.B., et al. (2023). Brain expression quantitative trait locus and network analyses reveal downstream effects and putative drivers for brain-related diseases. Nat. Genet. *55*, 377–388.
29. Sieberts, S.K., Perumal, T.M., Carrasquillo, M.M., Allen, M., Reddy, J.S., Hoffman, G.E., Dang, K.K., Calley, J., Ebert, P.J., Eddy, J., et al. (2020). Large eQTL meta-analysis reveals differing patterns between cerebral cortical and cerebellar brain regions. Sci. Data *7*, 340–411.
30. Storey, J.D., and Tibshirani, R. (2003). Statistical significance for genomewide studies. Proc. Natl. Acad. Sci. USA *100*, 9440–9445.
31. Gusev, A., Ko, A., Shi, H., Bhatia, G., Chung, W., Penninx, B.W.J.H., Jansen, R., de Geus, E.J.C., Boomsma, D.I., Wright, F.A., et al. (2016). Integrative approaches for large-scale transcriptome-wide association studies. Nat. Genet. *48*, 245–252.
32. Caggiano, C., Boudaie, A., Shemirani, R., Mefford, J., Petter, E., Chiu, A., Ercelen, D., He, R., Tward, D., Paul, K.C., et al. (2023). Health care utilization of fine-scale identity by descent clusters in a Los Angeles biobank. Nat. Med. *29*, 1845–1856.
33. Johnson, R., Ding, Y., Venkateswaran, V., Bhattacharya, A., Boulier, K., Chiu, A., Knyazev, S., Schwarz, T., Freund, M., Zhan, L., et al. (2022). Leveraging genomic diversity for discovery in an electronic health record linked biobank: the UCLA ATLAS Community Health Initiative. Genome Med. *14*, 104.
34. Wu, P., Gifford, A., Meng, X., Li, X., Campbell, H., Varley, T., Zhao, J., Carroll, R., Bastarache, L., Denny, J.C., et al. (2019). Mapping ICD-10 and ICD-10-CM Codes to Phecodes: Workflow Development and Initial Evaluation. JMIR Med. Inform. *7*, e14325.

35. Ritchie, M.E., Phipson, B., Wu, D., Hu, Y., Law, C.W., Shi, W., and Smyth, G.K. (2015). limma powers differential expression analyses for RNA-sequencing and microarray studies. Nucleic Acids Res. *43*, e47.
36. Chernecky, C.C., and Berger, B.J. (1997). Laboratory Tests and Diagnostic Procedures (W.B. Saunders Company).
37. George-Gay, B., and Parker, K. (2003). Understanding the complete blood count with differential. J. Perianesth. Nurs. *18*, 96–114.
38. Schmiedel, B.J., Gonzalez-Colin, C., Fajardo, V., Rocha, J., Madrigal, A., Ramírez-Suástegui, C., Bhattacharyya, S., Simon, H., Greenbaum, J.A., Peters, B., et al. (2022). Single-cell eQTL analysis of activated T cell subsets reveals activation and cell type-dependent effects of disease-risk variants. Sci. Immunol. *7*, eabm2508.
39. Chen, L., Ge, B., Casale, F.P., Vasquez, L., Kwan, T., Garrido-Martín, D., Watt, S., Yan, Y., Kundu, K., Ecker, S., et al. (2016). Genetic Drivers of Epigenetic and Transcriptional Variation in Human Immune Cells. Cell *167*, 1398–1414.e24.
40. Moffat, J.J., Ka, M., Jung, E.-M., Smith, A.L., and Kim, W.-Y. (2017). The role of MACF1 in nervous system development and maintenance. Semin. Cell Dev. Biol. *69*, 9–17.
41. Bryois, J., Calini, D., Macnair, W., Foo, L., Urich, E., Ortmann, W., Iglesias, V.A., Selvaraj, S., Nutma, E., Marzin, M., et al. (2022). Cell-type-specific cis-eQTLs in eight human brain cell types identify novel risk genes for psychiatric and neurological disorders. Nat. Neurosci. *25*, 1104–1112.
42. Westra, H.-J., Arends, D., Esko, T., Peters, M.J., Schurmann, C., Schramm, K., Kettunen, J., Yaghootkar, H., Fairfax, B.P., Andiappan, A.K., et al. (2015). Cell Specific eQTL Analysis without Sorting Cells. PLoS Genet. *11*, e1005223.
43. Schmiedel, B.J., Singh, D., Madrigal, A., Valdovino-Gonzalez, A.G., White, B.M., Zapardiel-Gonzalo, J., Ha, B., Altay, G., Greenbaum, J.A., McVicker, G., et al. (2018). Impact of Genetic Polymorphisms on Human Immune Cell Gene Expression. Cell *175*, 1701–1715.e16.
44. Kerimov, N., Hayhurst, J.D., Peikova, K., Manning, J.R., Walter, P., Kolberg, L., Samoviča, M., Sakthivel, M.P., Kuzmin, I., Trevanion, S.J., et al. (2021). A compendium of uniformly processed human gene expression and splicing quantitative trait loci. Nat. Genet. *53*, 1290–1299.
45. Yazar, S., Alquicira-Hernandez, J., Wing, K., Senabouth, A., Gordon, M.G., Andersen, S., Lu, Q., Rowson, A., Taylor, T.R.P., Clarke, L., et al. (2022). Single-cell eQTL mapping identifies cell type-specific genetic control of autoimmune disease. Science *376*, eabf3041.
46. Giambartolomei, C., Vukcevic, D., Schadt, E.E., Franke, L., Hingorani, A.D., Wallace, C., and Plagnol, V. (2014). Bayesian test for colocalisation between pairs of genetic association studies using summary statistics. PLoS Genet. *10*, e1004383.
47. Muthusamy, N., Chen, Y.-J., Yin, D.-M., Mei, L., and Bergson, C. (2015). Complementary roles of the neuron-enriched endosomal proteins NEEP21 and calcyon in neuronal vesicle trafficking. J. Neurochem. *132*, 20–31.
48. Najar, M.A., Rex, D.A.B., Modi, P.K., Agarwal, N., Dagamajalu, S., Karthikkeyan, G., Vijayakumar, M., Chatterjee, A., Sankar, U., and Prasad, T.S.K. (2021). A complete map of the Calcium/calmodulin-dependent protein kinase kinase 2 (CAMKK2) signaling pathway. J. Cell Commun. Signal. *15*, 283–290.
49. Farioli-Vecchioli, S., Micheli, L., Saraulli, D., Ceccarelli, M., Cannas, S., Scardigli, R., Leonardi, L., Cinà, I., Costanzi, M., Ciotti, M.T., et al. (2012). Btg1 is Required to Maintain the Pool of Stem and Progenitor Cells of the Dentate Gyrus and Subventricular Zone. Front. Neurosci. *6*, 124.
50. Mullins, N., Forstner, A.J., O'Connell, K.S., Coombes, B., Coleman, J.R.I., Qiao, Z., Als, T.D., Bigdeli, T.B., Børte, S., Bryois, J., et al. (2021). Genome-wide association study of more than 40,000 bipolar disorder cases provides new insights into the underlying biology. Nat. Genet. *53*, 817–829.
51. Consortium, T.S.W.G. of T.P.G., The Schizophrenia Working Group of the Psychiatric Genomics Consortium, Ripke, S., Walters, J.T.R., and O'Donovan, M.C. Mapping genomic loci prioritises genes and implicates synaptic biology in schizophrenia. Nature 604, 502-508.
52. Wray, N.R., Ripke, S., Mattheisen, M., Trzaskowski, M., Byrne, E.M., Abdellaoui, A., Adams, M.J., Agerbo, E., Air, T.M., Andlauer, T.M.F., et al. (2018). Genome-wide association analyses identify 44 risk variants and refine the genetic architecture of major depression. Nat. Genet. *50*, 668–681.
53. Walters, R.K., Polimanti, R., Johnson, E.C., McClintick, J.N., Adams, M.J., Adkins, A.E., Aliev, F., Bacanu, S.-A., Batzler, A., Bertelsen, S., et al. (2018). Transancestral GWAS of alcohol dependence reveals common genetic underpinnings with psychiatric disorders. Nat. Neurosci. *21*, 1656–1669.
54. Johnson, E.C., Demontis, D., Thorgeirsson, T.E., Walters, R.K., Polimanti, R., Hatoum, A.S., Sanchez-Roige, S., Paul, S.E., Wendt, F.R., Clarke, T.-K., et al. (2020). A large-scale genome-wide association study meta-analysis of cannabis use disorder. Lancet Psychiatr. *7*, 1032–1045.
55. Watanabe, K., Stringer, S., Frei, O., Mirkov, M.U., de Leeuw, C., Polderman, T.J.C., van der Sluis, S., Andreassen, O.A., Neale, B.M., and Posthuma, D. (2020). Author Correction: A global overview of pleiotropy and genetic architecture in complex traits. Nat. Genet. *52*, 353.
56. Jansen, P.R., Watanabe, K., Stringer, S., Skene, N., Bryois, J., Hammerschlag, A.R., de Leeuw, C.A., Benjamins, J.S., Muñoz-Manchado, A.B., Nagel, M., et al. (2019). Genome-wide analysis of insomnia in 1,331,010 individuals identifies new risk loci and functional pathways. Nat. Genet. *51*, 394–403.
57. Demontis, D., Walters, R.K., Martin, J., Mattheisen, M., Als, T.D., Agerbo, E., Baldursson, G., Belliveau, R., Bybjerg-Grauholm, J., Bækvad-Hansen, M., et al. (2019). Discovery of the first genome-wide significant risk loci for attention deficit/hyperactivity disorder. Nat. Genet. *51*, 63–75.
58. Jansen, I.E., Savage, J.E., Watanabe, K., Bryois, J., Williams, D.M., Steinberg, S., Sealock, J., Karlsson, I.K., Hägg, S., Athanasiu, L., et al. (2019). Genome-wide meta-analysis identifies new loci and functional pathways influencing Alzheimer's disease risk. Nat. Genet. *51*, 404–413.
59. PsychENCODE Consortium, Akbarian, S., Liu, C., Knowles, J.A., Vaccarino, F.M., Farnham, P.J., Crawford, G.E., Jaffe, A.E., Pinto, D., Dracheva, S., et al. (2015). The PsychENCODE project. Nat. Neurosci. *18*, 1707–1712.
60. Bentham, J., Morris, D.L., Graham, D.S.C., Pinder, C.L., Tombleson, P., Behrens, T.W., Martín, J., Fairfax, B.P., Knight, J.C., Chen, L., et al. (2015). Genetic association analyses implicate aberrant regulation of innate and adaptive immunity genes in the pathogenesis of systemic lupus erythematosus. Nat. Genet. *47*, 1457–1464.
61. van der Harst, P., Zhang, W., Mateo Leach, I., Rendon, A., Verweij, N., Sehmi, J., Paul, D.S., Elling, U., Allayee, H., Li, X., et al. (2012). Seventy-five genetic loci influencing the human red blood cell. Nature *492*, 369–375.

62. Bycroft, C., Freeman, C., Petkova, D., Band, G., Elliott, L.T., Sharp, K., Motyer, A., Vukcevic, D., Delaneau, O., O'Connell, J., et al. (2018). The UK Biobank resource with deep phenotyping and genomic data. Nature *562*, 203–209.
63. Wang, Y.-F., Zhang, Y., Lin, Z., Zhang, H., Wang, T.-Y., Cao, Y., Morris, D.L., Sheng, Y., Yin, X., Zhong, S.-L., et al. (2021). Identification of 38 novel loci for systemic lupus erythematosus and genetic heterogeneity between ancestral groups. Nat. Commun. *12*, 772.
64. Eames, H.L., Corbin, A.L., and Udalova, I.A. (2016). Interferon regulatory factor 5 in human autoimmunity and murine models of autoimmune disease. Transl. Res. *167*, 167–182.
65. Akkouh, I.A., Skrede, S., Holmgren, A., Ersland, K.M., Hansson, L., Bahrami, S., Andreassen, O.A., Steen, V.M., Djurovic, S., and Hughes, T. (2020). Exploring lithium's transcriptional mechanisms of action in bipolar disorder: a multi-step study. Neuropsychopharmacology *45*, 947–955.
66. Krebs, C.E., Ori, A.P.S., Vreeker, A., Wu, T., Cantor, R.M., Boks, M.P.M., Kahn, R.S., Olde Loohuis, L.M., and Ophoff, R.A. (2020). Whole blood transcriptome analysis in bipolar disorder reveals strong lithium effect. Psychol. Med. *50*, 2575–2586.
67. Zhou, W., Kanai, M., Wu, K.-H.H., Rasheed, H., Tsuo, K., Hirbo, J.B., Wang, Y., Bhattacharya, A., Zhao, H., Namba, S., et al. (2022). Global Biobank Meta-analysis Initiative: Powering genetic discovery across human disease. Cell Genom. *2*, 100192.
68. Smyth, G.K. limma: Linear Models for Microarray Data. Bioinformatics and Computational Biology Solutions Using R and Bioconductor 397–420.
69. Munkholm, K., Peijs, L., Vinberg, M., and Kessing, L.V. (2015). A composite peripheral blood gene expression measure as a potential diagnostic biomarker in bipolar disorder. Transl. Psychiatry *5*, e614.
70. Liharska, L.E., Park, Y.J., Ziafat, K., Wilkins, L., Silk, H., Linares, L.M., Thompson, R.C., Vornholt, E., Sullivan, B., Cohen, V., et al. (2023). A study of gene expression in the living human brain. Preprint at medRxiv. https://doi.org/10.1101/2023.04.21.23288916.
71. Lisé, M.F., Srivastava, D.P., Arstikaitis, P., Lett, R.L., Sheta, R., Viswanathan, V., Penzes, P., O'Connor, T.P., and El-Husseini, A. (2009). Myosin-Va-interacting protein, RILPL2, controls cell shape and neuronal morphogenesis via Rac signaling. J. Cell Sci. *122*, 3810–3821.
72. XY Ling, N., Langendorf, C.G., Hoque, A., Galic, S., Loh, K., Kemp, B.E., Gundlach, A.L., Oakhill, J.S., and Scott, J.W. (2020). Functional analysis of an R311C variant of Ca $^{2}$ -calmodulin-dependent protein kinase kinase-2 (CaMKK2) found as a de novo mutation in a patient with bipolar disorder. Bipolar Disord. *22*, 841–848.
73. Alda, M. (2015). LITHIUM IN THE TREATMENT OF BIPOLAR DISORDER: PHARMACOLOGY AND PHARMACOGENETICS. Mol. Psychiatry *20*, 661–670.
74. Grimes, C.A., and Jope, R.S. (2001). CREB DNA binding activity is inhibited by glycogen synthase kinase-3 beta and facilitated by lithium. J. Neurochem. *78*, 1219–1232.
75. Sakamoto, K., Karelina, K., and Obrietan, K. (2011). CREB: a multifaceted regulator of neuronal plasticity and protection. J. Neurochem. *116*, 1–9.
76. Karege, F., Perroud, N., Schürhoff, F., Méary, A., Marillier, G., Burkhardt, S., Ballmann, E., Fernandez, R., Jamain, S., Leboyer, M., et al. (2010). Association of AKT1 gene variants and protein expression in both schizophrenia and bipolar disorder. Genes Brain Behav. *9*, 503–511.
77. GTEx Consortium; Laboratory, Data Analysis & Coordinating Center LDACC—Analysis Working Group; Statistical Methods groups—Analysis Working Group; Enhancing GTEx eGTEx groups; NIH Common Fund; NIH/NCI; NIH/NHGRI; NIH/NIMH; NIH/NIDA; and Biospecimen Collection Source Site—NDRI (2017). Genetic effects on gene expression across human tissues. Nature *550*, 204–213.

The American Journal of Human Genetics, Volume *111*

## Supplemental information

# Cell-type deconvolution of bulk-blood RNA-seq reveals biological insights into neuropsychiatric disorders

**Toni Boltz, Tommer Schwarz, Merel Bot, Kangcheng Hou, Christa Caggiano, Sandra Lapinska, Chenda Duan, Marco P. Boks, Rene S. Kahn, Noah Zaitlen, Bogdan Pasaniuc, and Roel Ophoff**

## Supplemental Figures

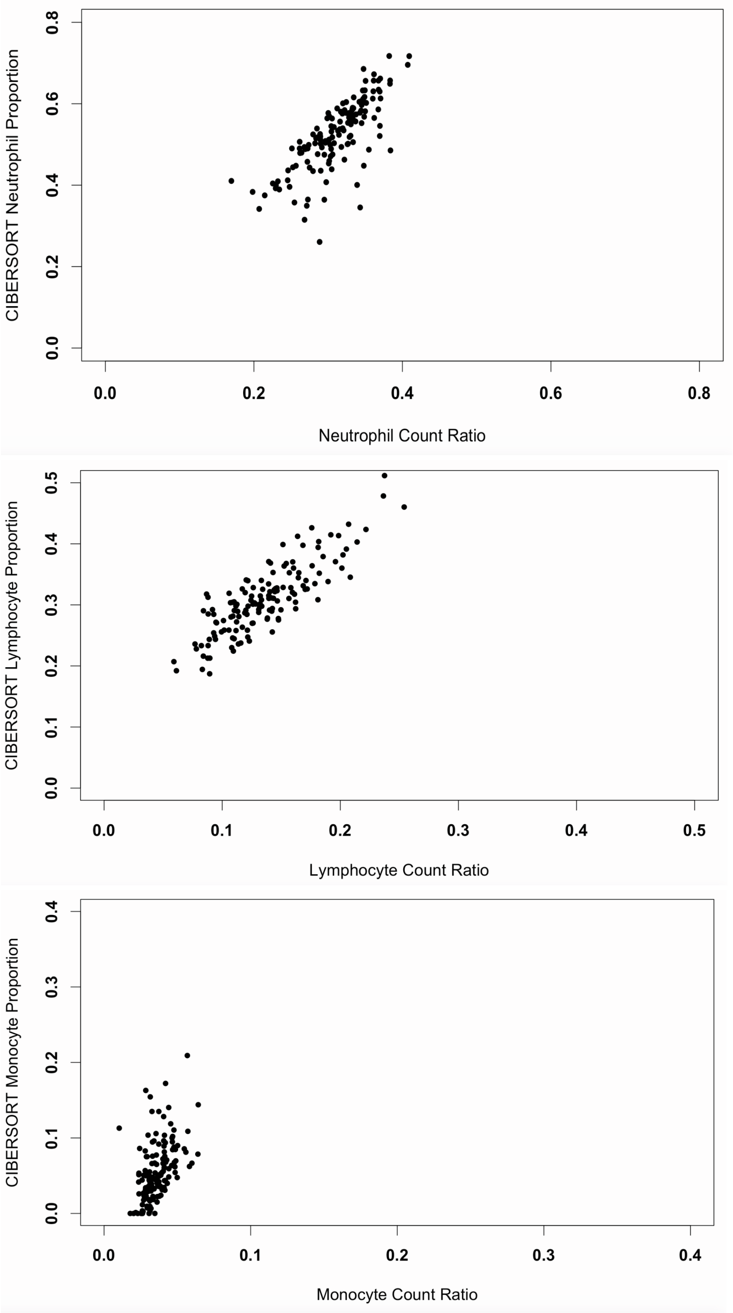


**Figure S1: Scatterplots of CIBERSORTx-estimated cell type proportions vs complete blood count proportions.** We find generally high concordance between computationally estimated and measured ground truth cell type proportions using a subset of our cohort. Pearson's correlation R2 for neutrophils = 0.76, for lymphocytes = 0.85, for monocytes = 0.48.

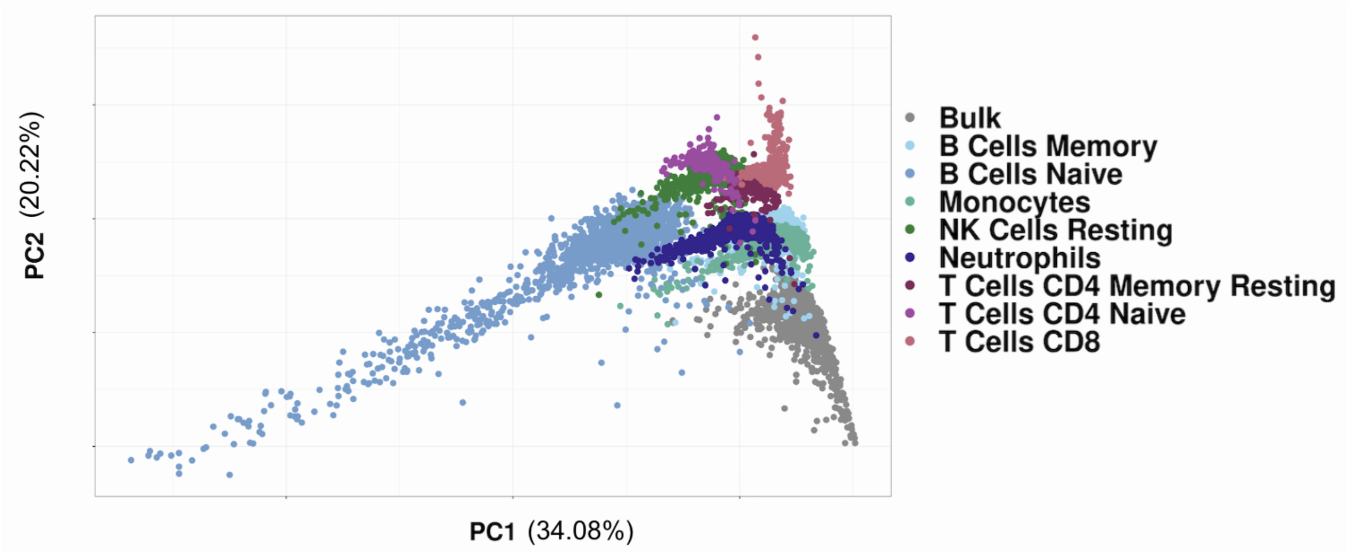


**Figure S2: Principal component analysis of cell type expression.** Each point represents an individual's gene expression profile, colored by the specific context, thus each individual is represented nine times on this plot.

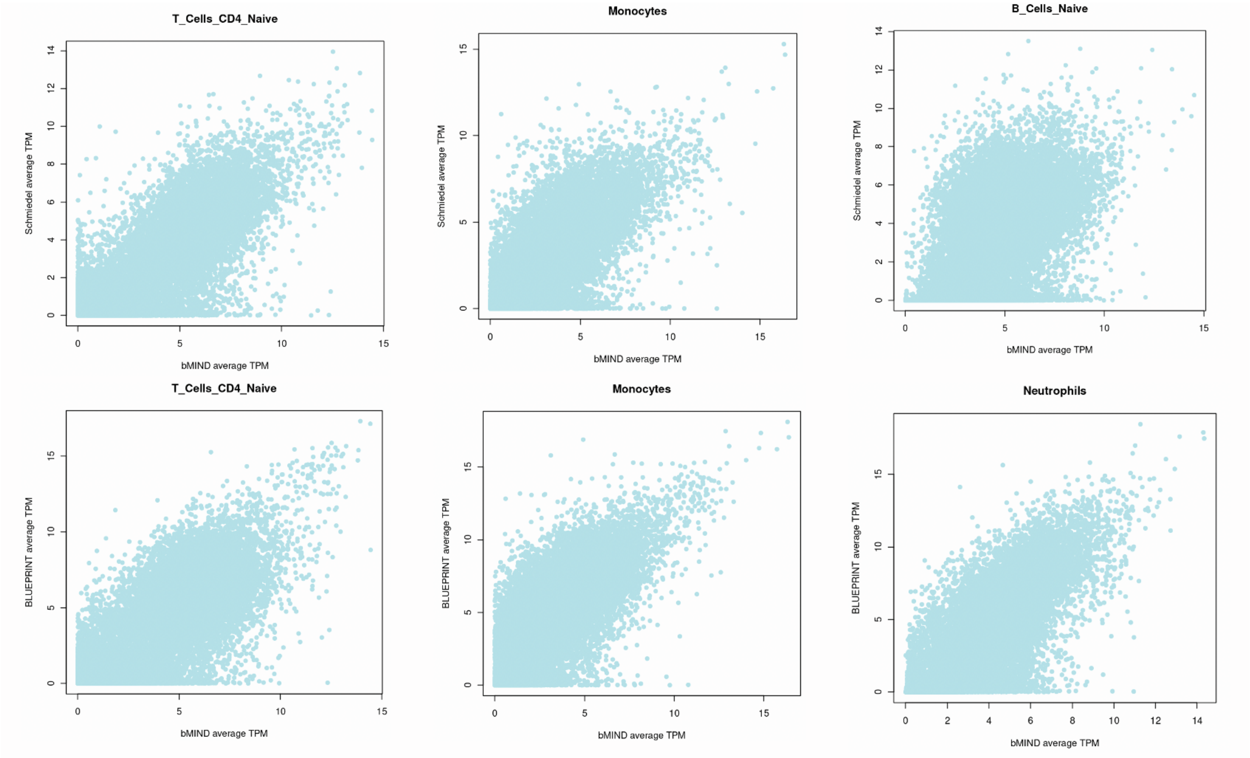


**Figure S3: Scatterplots of expression estimated from bulk vs single-cell reference datasets.**

Using two scRNA-Seq datasets as references (from the eQTLGen[1] study using BLUEPRINT[2] Consortium and Schmiedel et. al. (2018)[3] datasets, available online at: https://github.com/eQTL-Catalogue/eQTL-Catalogue-resources/blob/master/tabix/tabix_ftp_paths.tsv) we compare the median TPM values for protein coding genes using both scRNA-Seq and computationally deconvoluted bulk RNA-Seq.

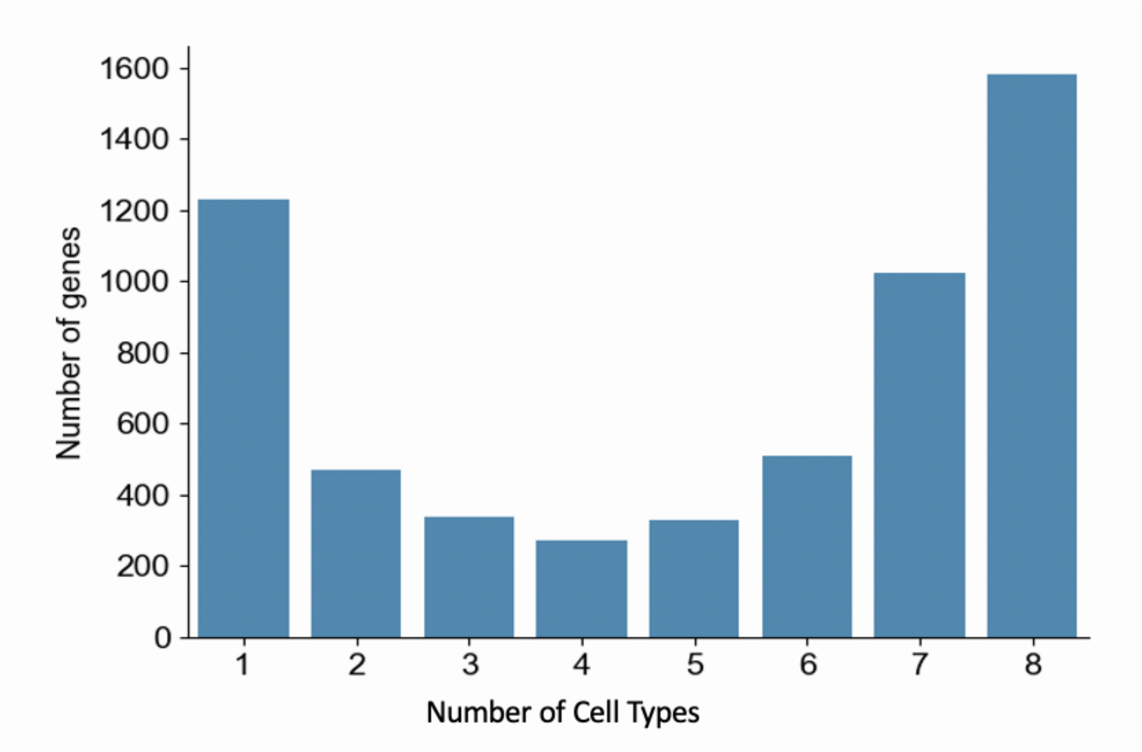


**Figure S4: Distribution of shared eGenes across cell type contexts.**

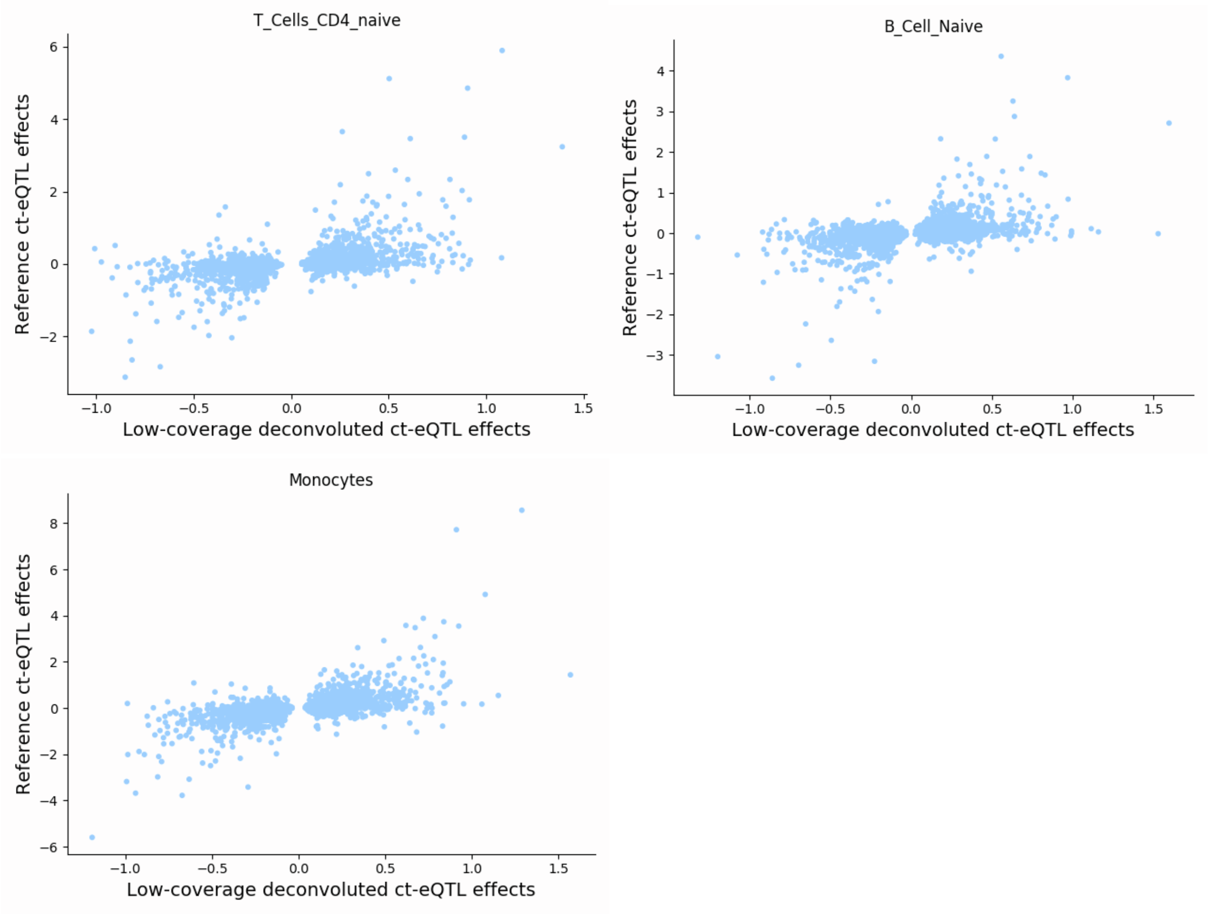


**Figure S5: Effect size correlations between reference single cell eQTL and the deconvoluted eQTL.** T cells CD4 naive R2 = 0.27; B cells naive R2 = 0.22; Monocytes R2 = 0.36.

**A.**

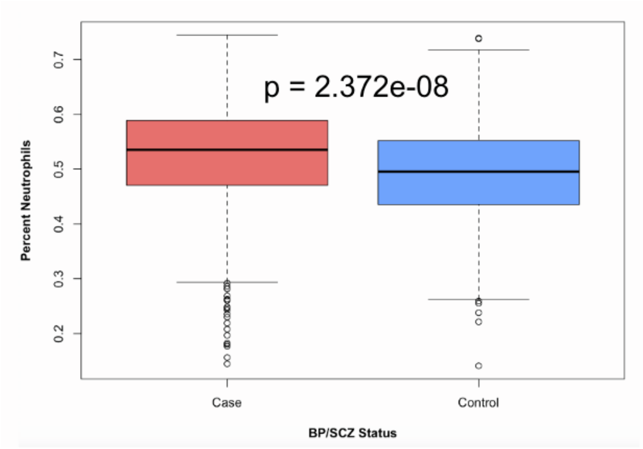


**B.**

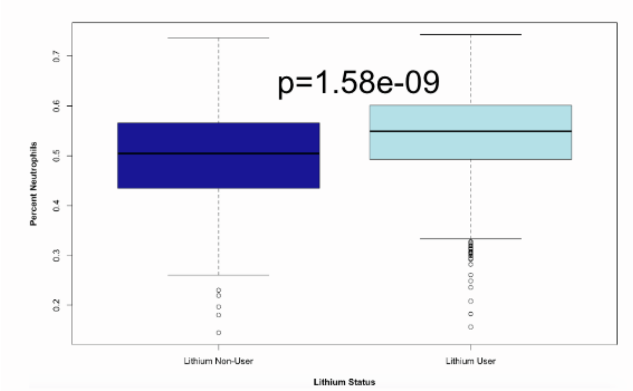


**C.**

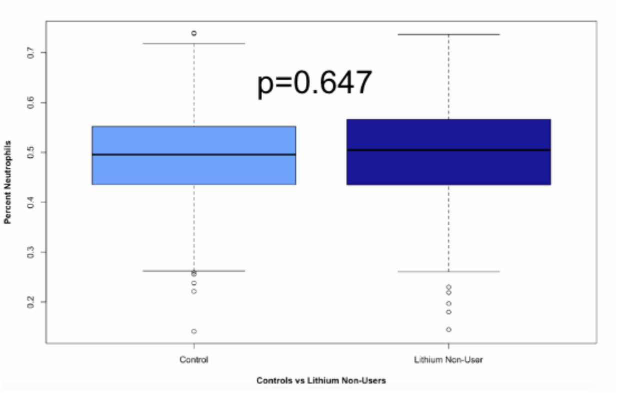


**Figure S6: Neutrophil count elevated for lithium users.**

A. Difference in neutrophil proportion (after accounting for covariates including age, sex, RIN, and RNA concentration) between BP/SCZ cases and controls.
B. Difference in neutrophil proportion between lithium users and non-users (after accounting for covariates), only within BP cases.
C. Difference in neutrophil proportion between lithium non-users and controls (after accounting for covariates).

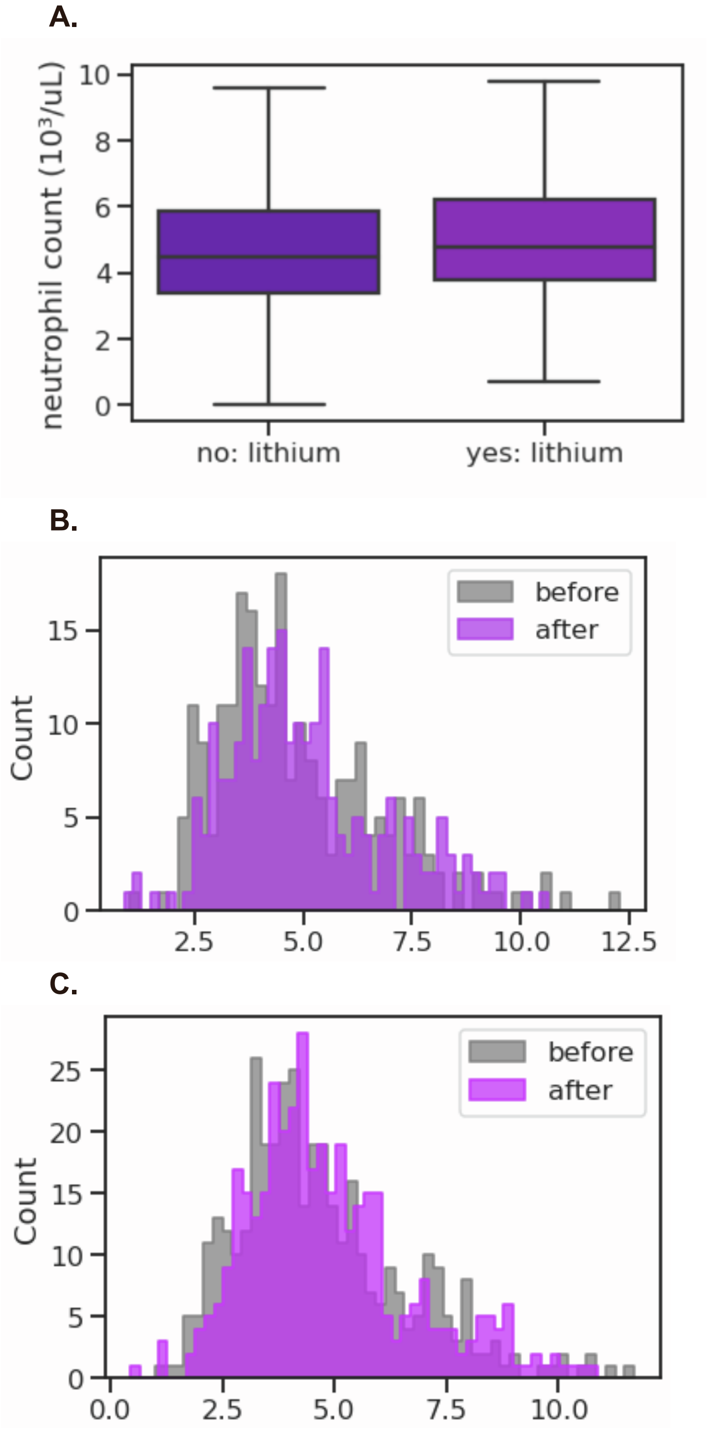


**Figure S7: Neutrophil count elevated for lithium users in UCLA ATLAS.**

A. Median neutrophil count across self-reported European individuals (N=7,350), with covariate correction for age and sex (p=2.09e-7).

B. Neutrophil count distribution across self-reported European individuals (n=229) before and after lithium prescription (Wilcoxon p=0.2).

C. Neutrophil count distribution across individuals (n=376, all ancestries) before and after lithium prescription (Wilcoxon p=0.0228).

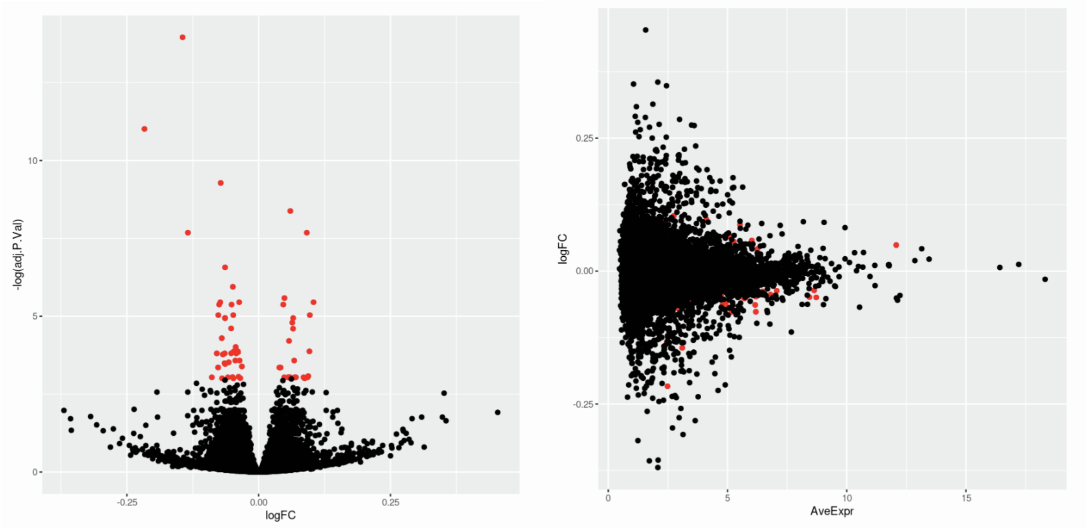


**Figure S8**: **Differential gene expression results for BP or SCZ cases vs controls:** (left) Volcano plot which highlights differentially expressed genes (FDR < 0.05) in red (N=64 total differentially expressed genes). (right) Average expression of each gene vs the log fold change (logFC) of each gene, with differentially expressed genes highlighted in red.

## Supplemental References


1. Kerimov, N., Hayhurst, J.D., Peikova, K., Manning, J.R., Walter, P., Kolberg, L., Samoviča, M., Sakthivel, M.P., Kuzmin, I., Trevanion, S.J., et al. (2021). A compendium of uniformly processed human gene expression and splicing quantitative trait loci. Nat. Genet. *53*, 1290–1299.

2. Chen, L., Ge, B., Casale, F.P., Vasquez, L., Kwan, T., Garrido-Martín, D., Watt, S., Yan, Y., Kundu, K., Ecker, S., et al. (2016). Genetic Drivers of Epigenetic and Transcriptional Variation in Human Immune Cells. Cell *167*, 1398–1414.e24.

3. Schmiedel, B.J., Gonzalez-Colin, C., Fajardo, V., Rocha, J., Madrigal, A., Ramírez-Suástegui, C., Bhattacharyya, S., Simon, H., Greenbaum, J.A., Peters, B., et al. (2022). Single-cell eQTL analysis of activated T cell subsets reveals activation and cell type-dependent effects of disease-risk variants. Science Immunology 7(68).
